# Veterinary antimicrobial use legislation: A comparative policy analysis of Kenya and Denmark

**DOI:** 10.1016/j.onehlt.2025.101260

**Published:** 2025-10-30

**Authors:** Simon Mbugua, Amos Lucky Mhone, Alexina Morang'a, Peter Gathura, Joshua Onono, Dishon M. Muloi, Arshnee Moodley

**Affiliations:** a Health Program, International Livestock Research Institute, Nairobi, Kenya; b Department of Public Health, Pharmacology and Toxicology, Faculty of Veterinary Medicine, University of Nairobi, Nairobi, Kenya; c Department of Veterinary and Animal Sciences, Faculty of Health and Medical Sciences, University of Copenhagen, Frederiksberg C, Denmark; d Institute of Infection, Veterinary and Ecological Sciences, University of Liverpool, Liverpool, United Kingdom

**Keywords:** Antimicrobial use, Legislation, Policies, Governance, Gap

## Abstract

Misuse of antimicrobial in both animal and human health remains a key driver of antimicrobial resistance (AMR). Strengthening policy and regulation framework is essential to promote responsible antimicrobial use (AMU). This study aimed to 1) identify policies and regulations governing AMU in Kenya's veterinary sector and 2) undertake a comparative analysis with Denmark's AMU governance system, widely recognised for its comprehensive approach, to identify potential areas for policy improvement. A desk-based review was conducted to identify relevant AMU policies and legislation in Kenya and Denmark. Data sources included the Food and Agriculture Organisation of the United Nations (FAO) databases (FAOLEX and AMR-LEX), government websites, and publications. Semi-structured interviews with key stakeholders from both countries were used to validate the findings. FAO's framework for analysing AMR-relevant legislation in the food and agriculture sector was used to analyse policies across key areas: veterinary medicinal products, animal health and production, feed legislation, pesticides, water quality, plant health, food safety, environmental health, soil and waste, and institutional coordination. We identified 547 legislative documents in Kenya and 206 in Denmark. The comparative analysis identified several areas where Kenya's policy framework could be further strengthened including enhanced regulation of the use of critically important antimicrobials for humans in animals, creation of sub-national AMU monitoring programs, definition of veterinary prescription benchmarks, and enforcement of restrictions on non-therapeutic use. Further, gaps were identified in policies addressing antimicrobial monitoring in the environment, particularly in soil and waste management, alongside the absence of a dedicated funding mechanism for the country's AMR coordination body and the lack of defined targets for reducing antimicrobial use in animals. The study highlights the importance of continued legislative development in Kenya to enhance AMU governance, particularly in the areas of veterinary oversight, monitoring, and environmental protection. Demark's experience illustrates the value of integrated legislative approach, which has contributed to measurable reductions in veterinary AMU. Tailoring and strengthening Kenya's regulatory framework, while ensuring coordinated institutional support and sustainable funding, can help align its AMR governance with international standards and contribute to more effective AMR mitigation efforts.

## 1 Introduction

Misuse of antimicrobials remains a key driver of antimicrobial resistance (AMR), with disproportionate burden borne by low- and middle-income countries (LMICs), where inadequate stewardship and regulatory frameworks have further exacerbated the crisis [1,2]. By 2030, antimicrobial sales are projected to increase globally by 11.5 %, largely driven by demand in LMICs [3]. Studies have demonstrated widespread misuse of antimicrobials across various LMICs, including Kenya [4]. In animal husbandry, an estimated 73 % of global antimicrobial use (AMU) occurs in healthy animals for prophylaxis and growth promotion, practices that substantially accelerate the emergence and spread of AMR across humans, animals, and the environment [5]. Research in Kenya has demonstrated bacteria isolated from animals exhibiting resistance even to critical antibiotics in human medicine [6,7]. Antimicrobial misuse can be attributed to several factors, including weak and fragmented legislature [8].

The establishment of clear, enforceable legislation is essential in addressing AMR challenges [9]. Effective legislative frameworks typically focus on regulating importation and distribution of antimicrobials [10], restricting the use of medically important antimicrobials [11], enhancing surveillance systems, promoting the development of alternative treatments, and encouraging best practices in animal husbandry [12]. Globally, the regulatory landscape for veterinary AMU advocates for a harmonised approach in both human and veterinary medicine to prevent the spread of resistant pathogens [13]. Regulatory frameworks in high-income countries, such as Denmark, are characterized by strong enforcement mechanisms, comprehensive surveillance systems, and a high level of public and political awareness about AMR [14]. These frameworks are further supported by robust veterinary infrastructure and availability of alternative disease prevention strategies, such as vaccines and improved biosecurity measures [15]. In contrast, the regulation of veterinary AMU in LMICs is often underdeveloped, presenting unique challenges to controlling AMR. LMICs are characterized by livestock production systems that often are driven by the need for food security and economic development [16]. In many of these countries, antimicrobials are used not only for therapeutic purposes but also for growth promotion and disease prevention, often with absence of robust biosecurity practices [17].

Over-the-counter availability of antimicrobials without prescription, and easy access in Kenya contributes to their widespread overuse and misuse [18,19]. This is further exacerbated by a lack of public awareness about AMR coupled with limited access to veterinary services [20,21]. Economic and infrastructural barriers such as limited financial resources, a shortage of public veterinary professionals, weak surveillance systems, and insufficient resources for monitoring and enforcing compliance, hinders effective AMU governance [22]. Moreover, significant legislative gaps persist, often due to inconsistent guidelines or the absence of comprehensive policies addressing AMU across agricultural sectors. This study aimed to (a) conduct a gap analysis in Kenya's AMU-related policies and regulations, and (b) compare them to the Danish AMU regulations. Denmark was used as a reference country because of its well-established, evidence-based approach to AMU management in animal health. Over several decades, the country has implemented integrated monitoring, veterinary oversight, and data-driven policies that have led to measurable reductions in AMU. Between 2014 and 2023, Denmark achieved a 20 % reduction in veterinary AMU and consistently reports lower levels of antimicrobial use and resistance of animal origin compared with most other EU countries [23]. The comparison is intended to highlight opportunities for contextual adaptation, rather than direct replication, recognising Kenya's ongoing progress in strengthening AMU governance within a rapidly evolving livestock sector.

## 2 Material and methods

### 2.1 Study design

A desk-based literature review was conducted to collect and synthesize existing data on AMU legislation. This approach enabled a comparison between Kenyan regulations and those of Denmark.

#### 2.1.1 Search strategy

The review utilized comprehensive searches on (a) academic databases namely Google Scholar and PubMed, (b) government websites namely the Danish Ministry of Food, Agriculture, and Fisheries, and the Kenyan Ministry of Agriculture, Livestock, and Fisheries (MALF), and (c) the Food and Agriculture Organisation of the United Nations (FAO) FAOLEX and AMR-LEX databases. FAOLEX is a global repository of national laws, regulations, and policies related to food, agriculture, and natural resources. Maintained by the FAO's Development Law Service, it offers free access to abstracts, indexing information, and in many cases, full-text legal documents from around the world [24]. Building on this foundation, AMR-LEX was launched by FAO in September 2022. AMR-LEX is a publicly accessible legal database that specifically focuses on legal and policy instruments related to AMR and AMU in the food and agriculture sectors. It adopts a One Health approach and includes both AMR-specific legislation and broader sectoral laws that influence AMU. The platform also provides country and regional profiles, enabling users to explore regulatory landscapes and compare approaches across contexts [25].

The search focused on grey literature, government gazettes, and official publications, guided by keywords including “Kenya AMU policies”, “Denmark AMU policies”, “legislation”, “laws”, “strategy”, “governance”, and “regulation”. Documents were included if they provided information on AMU policies, relevant laws and acts, or described the roles of regulatory bodies. Materials from both Kenya and Denmark were reviewed to ensure comprehensive coverage. Data extraction focused on key aspects such as policy content, regulatory bodies involved, publication years, establishment dates, and subsequent amendments. Danish documents were translated into English to ensure consistency during the analysis.

#### 2.1.2 Document analysis

The FAO methodology for analysing AMR-relevant legislation in the food and agriculture sector was used to evaluate policy documents. This methodology constitutes nine key areas of assessment: (a) veterinary medicinal products, (b) animal health and production practices to prevent animal diseases in terrestrial and aquatic animals, (c) feed registration, (d) pesticides, (e) food safety, (f) environment, soil and waste, (g) water quality, (h) plant health, and (i) institutional coordination [26]. A comparative analysis was conducted to identify gaps and areas for improvement in Kenya's AMU policies by benchmarking them against Denmark's policies. This data was organized into tables and visualized using Excel and R software.

#### 2.1.3 Stakeholder interviews

Semi-structured interviews with key stakeholders in Kenya and Denmark were conducted to validate findings from the literature review. Participants included representatives from relevant ministries, regulatory and policy institutions, such as Kenya's Directorate of Veterinary Services (DVS) and Veterinary Medicines Directorate (VMD), and the Danish Ministry of Food, Agriculture, and Fisheries. The interviews focused on the effectiveness of current laws, identifying gaps in policy enforcement, and highlighting areas for improvement. Audio recordings from the interviews were carefully reviewed, and relevant information was transcribed and transferred to NVIVO version 14.23.0 for thematic qualitative analysis.

## 3 Results

### 3.1 Mapping antimicrobial use legislation in the Kenyan and Danish agricultural sector

**Fig. 1:**
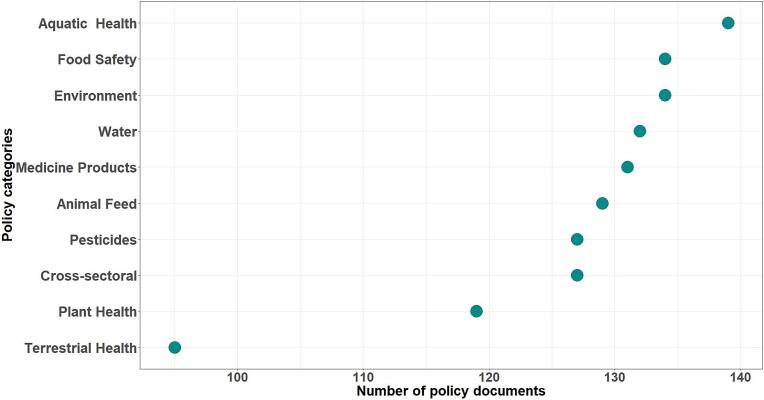
Categorization of laws related to antimicrobial use in the Kenyan agricultural sector.

A total of 547 sector-specific and cross-sectoral laws related to AMU were identified in Kenya's agricultural sector. Most of the laws addressed aquatic animal health (*n* = 139), environmental protection (*n* = 134), plant health (119), and food safety (n = 134), while fewer focused on terrestrial animal health (*n* = 95) (Fig. 1). In comparison, 206 legislative documents related to AMU were identified in Denmark's agricultural sector (Supplementary material 1).

### 3.2 Gaps in antimicrobial use legislation in the Kenyan agricultural sector

A comparison with Denmark's AMU regulatory framework (see Supplementary material 1) highlighted several areas where Kenya's legislative system could be strengthened, as summarized below.

#### 3.2.1 Veterinary medicinal products

Kenya's regulatory framework for distribution of veterinary antimicrobials presents opportunities for further development. Currently, there is no specific legislation restricting or authorizing the sale and use of antimicrobials classified as critically important for human health, such as colistin, in animal production. Similarly, there are no legal prohibitions on the use of antimicrobials for growth promotion. The Veterinary Medicines Directorate (VMD), Kenya's national medicine authority, requires cabinet-level or ministerial initiation for amendments to secondary regulations, a process that can limit timely responses to emerging scientific evidence; the last amendments were made in 2015. Furthermore, Kenya has yet to establish an essential veterinary medicines list (Table 1; Supplementary material 1).

**Table 1:** Summary of gaps identified in the antimicrobial use legislation in the Kenyan agricultural sector.

Category	Gap Identified
Veterinary medicinal products	• Lack of legislation restricting or authorizing the sale and use of critically important antimicrobials for human health (e.g., colistin) in animals. • No legal provisions specifically banning the use of antimicrobials for growth promotion in animals. • Limited flexibility for regulatory updates • Absence of a national list of essential veterinary medicines
Animal Health and Production	• No restrictions on the use of manure from antimicrobial treated animals as fertilizer. • No guidelines on disposal of animals treated with antimicrobials and their by-products. • Lack of provisions for managing wastewater and waste from cleaning premises where animals treated with antimicrobials were housed.
Medicated Feed	• No restrictions on non-therapeutic use of medicated feed or the use of antibiotic growth promoters
Environment, soil, and waste	• No national standards for monitoring antimicrobial residues in soil. • No provisions for managing antimicrobial waste and effluents.
Institutional Coordination	• No legal framework for allocating funding to the National Antimicrobial Stewardship Interagency Committee (NASIC) or County Antimicrobial Stewardship Interagency Committees (CASICs) • Absence of a prioritized research agenda on AMR
The Role of Herd Veterinarians	• Lack of host-specific or disease-specific treatment guidelines • Unclear regulations on veterinarian sales of veterinary medicines • No mandatory veterinary advisory service contracts • No legal mandate for unannounced farm inspections or veterinarians farm revisits
Antimicrobial Use Monitoring Program	• No budget allocation for AMU monitoring • No national AMU reduction targets in animals • Lack of benchmarking system for veterinary antibiotic prescriptions. • Diagnostic testing not mandated • No requirement for farmer training on best farming practices and responsible AMU.

#### 3.2.2 Animal health and production

While Kenya has developed guidelines promoting the prudent use of antimicrobials, gaps remain in addressing the management of waste from treated animals. Current guidelines do not restrict the use of manure from treated animals as crop fertilizer, nor do they provide specific measures for the disposal of antimicrobial-treated animals, their by-products, or effluents and waste that result from cleaning the premises of animals treated with AMs (Table 1) (Supplementary material 1).

#### 3.2.3 Medicated feed

Kenya's regulatory framework for medicated feed also presents opportunities for further development. There are currently no legal restrictions on the non-therapeutic sale and use of medicated feed, including the use of antibiotics as growth promoters (Table 1; Supplementary material 1).

#### 3.2.4 Environment, soil, and waste

Environmental legislation in Kenya could be strengthened to more effectively manage the risks posed by antimicrobial-related waste. National standards for monitoring antimicrobial residues in soil and specific provisions for controlling waste and effluents from mills, industries and farms are currently lacking (Table 1; Supplementary material 1).

### 3.3 Institutional coordination

The National Antimicrobial Stewardship Interagency Committee (NASIC) and County Antimicrobial Stewardship Interagency Committees (CASICs) coordinate Kenya's AMR efforts at the national and county levels. However, there is no legal framework ensuring dedicated funding for these bodies to implement activities outlined in NAP. Additionally, the absence of a prioritized research agenda on AMR limits evidence generation needed to inform future policy decisions (Table 1; Supplementary material 1).

#### 3.3.1 The role of herd veterinarians

Opportunities exist to improve veterinary oversight. Current legislation does not provide specific treatment guidelines for different animal species or diseases, nor does it clearly regulate whether veterinarians may sell veterinary products for profit. Farms are not required to maintain veterinary advisory service contracts, which may weaken accountability. Furthermore, there is no mandate for unannounced farm inspections, and veterinarians are not obligated to revisit farms when deficiencies have been identified. (Table 1; Supplementary material 1).

### 3.4 Antimicrobial use monitoring program

Kenya's legal framework for AMU monitoring presents several opportunities for strengthening. At present, there are no provisions for dedicated budget allocations to support national AMU monitoring, nor defined national targets for reducing AMU in animals. A system for benchmarking veterinary antibiotic prescriptions in lacking, and diagnostic testing prior to treatment is not mandated. Finally, farmers are not required to attend training on best farming practices, biosecurity, or responsible AMU, which could support regulatory compliance and stewardship efforts (Table 1; Supplementary material 1).

## 4 Discussion

Restricting access to antimicrobials remains a critical strategy for reducing overall AMU and curtailing the rise of AMR. In Kenya, there is an important opportunity to strengthen legislation by introducing specific provisions governing the use of antimicrobials critically important for human health in animals. The continued availability and use of such antibiotics in food-producing animals pose a risk to human health, as shown by studies reporting bacterial resistance to these antibiotics in animals in Kenya [6,7]. Addressing this gap through targeted regulatory measures would be instrumental in safeguarding the efficacy of life-saving antimicrobials.

The non-therapeutic use of antimicrobials, particularly as additives in feed or water for growth promotion, remains unregulated in Kenya. As in many LMICs, the use of antibiotic feed additives to improve productivity contributes significantly to the acceleration of AMR [27]. However, this challenge also presents a timely opportunity for reform. Global momentum is building around this issue, as emphasized by the 2024 United Nations General Assembly High-Level Meeting on AMR, where member states endorsed a political declaration calling for urgent legislative action [28]. Evidence shows that interventions restricting AMU resulted in measurable reductions AMR prevalence among animals [[29], [30], [31], [32]]. Countries such as Namibia [33], Denmark [15], and several other European countries [34] have demonstrated the effectiveness of such bans, some of which have been in place since 1991. Kenya is well positioned to align with these best practices to protect public, animal and environmental health.

Currently, Kenya's AMU Surveillance System for Antimicrobial Consumption (KESAC) [35] is limited the human health sector, with no coverage of AMU in the veterinary or agricultural sectors. Expanding surveillance efforts to encompass the veterinary and agricultural sectors is crucial. Establishing a comprehensive AMU monitoring system, including data collection on antimicrobial imports, sales, and usage, and setting national AMU reduction targets, would support transparency and accountability across the antimicrobial supply chain [36,37]. Denmark's Integrated Antimicrobial Resistance Monitoring and Research Program (DANMAP) [23]integrated data systems that support evidence-based policymaking.

Kenya has made progress toward improved animal health governance, notably through the 2021 Animal Health Bill currently under parliamentary review [38]. This Bill, which addresses animal identification, traceability, feed regulation, and veterinary diagnostics, represents a positive step toward alignment World Organisation for Animal Health's (WOAH) Animal Health Code [39]. However, complementary reforms are needed. In particular, the absence of a benchmarking system to monitor veterinarians' prescribing patterns remain a significant gap. Systems such as Denmark's VETSTAT database have successfully promoted responsible AMU through data-driven oversight [40].

The National Antimicrobial Stewardship Interagency Committee (NASIC) plays a key role in Kenya's multi-sectorial response to AMR [41]. However, NASIC operations are constrained by a lack of dedicated and sustainable funding, relying heavily on donor support and periodic government allocations [42,43]. Establishing a consistent funding mechanism through budgetary provisions or public-private partnerships would enhance NASIC's capacity to implement and sustain AMR initiatives. Additionally, the VMD currently lacks the autonomy to amend secondary legislation in response to emerging scientific evidence; such amendments must be initiated by the Cabinet Secretary for Agriculture, the VMD Board, or the Attorney General. This centralized process limits the agility of regulatory updates needed to reflect evolving evidence and international best practices. Strengthening the VMD to revise regulations based on emerging evidence would enhance the agility and effectiveness of Kenya's AMR response and ensure alignment with international best practices [44].

Kenya's environmental legislation could also be strengthened by incorporating national standards for monitoring antimicrobial residues in soil and regulating the disposal of antimicrobial-contaminated waste from agricultural operations. Addressing these gaps is critical for safeguarding water and soil quality, protecting ecosystem health while reinforcing Kenya's broader commitment to responsible antimicrobial stewardship [45,46].

The Guidelines for Prudent Use of Antimicrobials in Animals in Kenya provide a valuable starting point for antimicrobial stewardship [47]. However, the absence of standard treatment guidelines, an essential veterinary medicines list, and mandatory diagnostic testing to inform treatment decisions limits their impact. In the absence of these critical tools, many animal health practitioners continue to rely on empirical treatment. Studies have shown that antibiotics are frequently prescribed without proper diagnosis or a clear understanding of the disease status of animals [44], leading to widespread misuse. Weak enforcement, evident in the lack of penalties for non-compliance and absence of farm re-visits, further encourages inappropriate AMU practices [48]. These findings are supported by various studies reporting widespread antibiotic misuse on farms due to inadequate veterinary supervision [[49], [50], [51]]. The Danish “Yellow Card” system was introduced in 2010 to reduce the use of antimicrobials in pig production, and it targets farms with high consumption and holds the farmer accountable [52]. If a farm's average antimicrobial consumption over a period exceeds the given threshold, the Danish Veterinary and Food Administration issues an order compelling the farm owner to reduce AMU below the threshold within nine months. The system was further developed by assigning weights to certain antibiotic classes i.e. assigning greater weight to those antibiotics critically for human health, e.g. colistin, fluoroquinolones and cephalosporins, thereby discouraging their use. This differentiated model has been effective and since 2014, the overall usage of antimicrobials in animals decreased annually and total consumption was 20 % lower in 2023 compared to 2014 [23]. Implementing such a model in Kenya would require granular AMU monitoring at the farm level but could substantially improve stewardship and accountability.

The 2024 United Nations General Assembly High Level Meeting political declaration on AMR reaffirms the urgency of legislative and governance reaffirms, emphasizing multisectoral action, sustainable financing, and a One Health approach [28].As a UN member state, Kenya commitments to the 2030 AMR goals offers a strong impetus for reform across key areas, including veterinary medicines regulation, AMU surveillance, environmental protections, and veterinary oversight. Prioritizing these reforms will not only protect human and animal health but also contribute to global efforts to address AMR in a sustainable and equitable manner. This study relied exclusively on publicly accessible online documents, which may not include all internal, unpublished or draft policies. As a result, some relevant legislative instruments or institutional practices were not included in the analysis, potentially limiting the comprehensiveness of the findings.

## 5 Conclusion

Kenya has made commendable progress in its fight against AMR, notably through the revision of the Animal Health Bill and the establishment of NASIC. However, critical legislative and institutional gaps remain, including unrestricted access to critically important antimicrobials, lack of regulations on non-therapeutic AMU, limited veterinary oversight, weak enforcement mechanisms, and the absence of an integrated AMU monitoring system covering the animal and environmental sectors. These challenges also present opportunities for constructive reform. Drawing on successful models, such as Denmark's VETSTAT and Yellow Card systems, Kenya can develop adaptive and evidence-based regulations. Strengthening veterinary legislation, establishing a robust AMU monitoring framework, introducing environmental safeguards, and securing sustainable funding for NASIC are essential steps. Crucially, Kenya's commitments under the 2024 UNGA High-Level Meeting on AMR offer both the impetus and the framework for action. By aligning national policies with these global goals, Kenya can enhance antimicrobial stewardship, protect public and animal health, and contribute meaningfully to global efforts to reduce AMR. With focused and timely reforms, the country has the potential to become a regional leader in responsible antimicrobial governance.

## CRediT authorship contribution statement

**Simon Mbugua:** Conceptualization, Data curation, Formal analysis, Investigation, Methodology, Software, Supervision, Validation, Visualization, Writing – original draft, Writing – review & editing. **Amos Lucky Mhone:** Data curation, Formal analysis, Investigation, Methodology, Project administration, Resources, Software, Validation, Visualization, Writing – original draft, Writing – review & editing. **Alexina Morang'a:** Writing – review & editing. **Peter Gathura:** Writing – review & editing. **Joshua Onono:** Writing – review & editing. **Dishon M. Muloi:** Conceptualization, Data curation, Formal analysis, Funding acquisition, Investigation, Methodology, Project administration, Resources, Software, Supervision, Validation, Visualization, Writing – original draft, Writing – review & editing. **Arshnee Moodley:** Methodology, Project administration, Resources, Software, Supervision, Validation, Visualization, Writing – original draft, Writing – review & editing, Conceptualization, Data curation, Formal analysis, Funding acquisition, Investigation.

## Ethics statement

Ethical approval for data collection was obtained from the International Livestock Research Institute (ILRI) Institutional Research Ethics Committee (ILRI-IREC2022–33). Written informed consent was obtained from all study participants.

## Funding

This study was funded by the 10.13039/501100006456German Federal Ministry for Economic Cooperation and Development (BMZ) through the One Health Research, Education and Outreach Centre in Africa (OHRECA) led by ILRI, the CGIAR One Health initiative “Protecting Human Health Through a One Health Approach”, and the CGIAR Science Programme *“Sustainable Animal and Aquatic Foods (SAAF)”* which was supported by contributors to the CGIAR Trust Fund (https://www.cgiar.org/funders/).

## Declaration of competing interest

The authors declare that they have no known competing financial interests or personal relationships that could have appeared to influence the work reported in this paper.

## Data Availability

Data will be made available on request.

## Appendix A Supplementary data

Supplementary material: Comparison of Kenya's and Denmark's legislation on veterinary antimicrobials and related products. Image 1
