# Supplementary material for: Veterinary antimicrobial use legislation: A comparative policy analysis of Kenya and Denmark
Source: One Health. 2025 Oct 30;21:101260. doi: 10.1016/j.onehlt.2025.101260 (PMC12630091; doi:10.1016/j.onehlt.2025.101260)
Supplement: Supplementary file 1 — Supplementary material: Comparison of Kenya's and Denmark's legislation on veterinary antimicrobials and related products. [file mmc1.docx]

**Supplementary Material 1**

|  | **Policy Review Description** | **Kenya** | **Kenya** | **Denmark** |  |
| --- | --- | --- | --- | --- | --- |
|  |  | **Status** | **Supporting Laws** | **Status** | **Supporting Laws** |
|  | **Veterinary medicinal products (Antimicrobials) national legislation** |  |  |  |  |
| **A** | **Definitions** |  |  |  |  |
| **1** | Is there a definition for VMP? Are VMPs part of a broader definition of medicinal products? | Yes | Veterinary Surgeons and Veterinary Paraprofessionals Act, 2015 (Veterinary Medicines Directorate Regulations)) | Yes | Veterinary medicinal products and repeal of Directive 2001/82/EC (VMP) (EU Regulation 2019/6 of 11 December 2018) (consolidated version of 28 January 2022);  Act on Medicinal Products (Lægemiddelloven, LBK nr 99 af 16/01/2024); Regulation (EU) 2019/6 on veterinary medicinal products |
| **2** | Is there a definition for AMs/AM agents or any other variations of this term? | Yes | Veterinary Surgeons and Veterinary Paraprofessionals Act, 2015 (Veterinary Medicines Directorate Regulations) | Yes | Veterinary medicinal products and repeal of Directive 2001/82/EC (VMP) (EU Regulation 2019/6 of 11 December 2018) (consolidated version of 28 January 2022)  Act on Medicinal Products (Lægemiddelloven, LBK nr 99 af 16/01/2024); Regulation (EU) 2019/6 on veterinary medicinal products |
|  |  |  |  |  |  |
| **B** | **Relevant authority** |  |  |  |  |
| **3** | Is there one designated authority in charge of authorizing and managing VMPs? | Veterinary Medicines Directorate | Veterinary Surgeons and Veterinary Paraprofessionals Act, 2015 (Veterinary Medicines Directorate Regulations) | The Danish Medicines Agency (Lægemiddelstyrelsen)  Danish Veterinary and Food Administration (DVFA) | Veterinary medicinal products and repeal of Directive 2001/82/EC (VMP) (EU Regulation 2019/6 of 11 December 2018) (consolidated version of 28 January 2022)  Act on Medicinal Products (Lægemiddelloven, LBK nr 99 af 16/01/2024 |
| **C** | **Authorization or registration of veterinary AMs** |  |  |  |  |
| **3** | Is there a government-approved registry of AMs or a list of AMs? | Yes | Veterinary Surgeons and Veterinary Paraprofessionals Act, 2015 (Veterinary Medicines Directorate Regulations) | Yes | Veterinary medicinal products and repeal of Directive 2001/82/EC (VMP) (EU Regulation 2019/6 of 11 December 2018) (consolidated version of 28 January 2022);Veterinarians' use, dispensing and prescribing, etc. of medicinal products for animals (Veterinary Executive Order) (BEK 646 of 31 May 2023);  Amendment Order to the Veterinary Executive Order (BEK 1149 of 4 September 2023);  Guidance on the Veterinary Medicinal Products Regulation and the Veterinary Executive Order.pdf  Act on Medicinal Products (Lægemiddelloven, LBK nr 99 af 16/01/2024); Executive Order No. 1234 of 2021 on Veterinary Medicinal Products; Regulation (EU) 2019/6 |
| **4** | Is there a law prohibiting the production, importation, distribution, supply, and use of veterinary medicinal products that have not been authorized/registered according to the national legislation? | Yes | Veterinary Surgeons and Veterinary Paraprofessionals Act, 2015 (Veterinary Medicines Directorate Regulations) | Yes | Veterinary medicinal products and repeal of Directive 2001/82/EC (VMP) (EU Regulation 2019/6 of 11 December 2018) (consolidated version of 28 January 2022); Veterinarians' use, dispensing and prescribing, etc. of medicinal products for animals (Veterinary Executive Order) (BEK 646 of 31 May 2023);  Amendment Order to the Veterinary Executive Order (BEK 1149 of 4 September 2023);  Guidance on the Veterinary Medicinal Products Regulation and the Veterinary Executive Order.pdf  Act on Medicinal Products (Lægemiddelloven, LBK nr 99 af 16/01/2024); Executive Order No. 1234 of 2021 on Veterinary Medicinal Products; Regulation (EU) 2019/6 |
| **5** | Is some reference or mechanism included in the legislation for transparency in decision-making? | Yes | Veterinary Surgeons and Veterinary Paraprofessionals Act, 2015 (Veterinary Medicines Directorate Regulations) | Yes | Veterinary medicinal products and repeal of Directive 2001/82/EC (VMP) (EU Regulation 2019/6 of 11 December 2018) (consolidated version of 28 January 2022); Veterinarians' use, dispensing and prescribing, etc. of medicinal products for animals (Veterinary Executive Order) (BEK 646 of 31 May 2023);  Amendment Order to the Veterinary Executive Order (BEK 1149 of 4 September 2023);  Guidance on the Veterinary Medicinal Products Regulation and the Veterinary Executive Order.pdf  Act on Medicinal Products (Lægemiddelloven, LBK nr 99 af 16/01/2024); Executive Order No. 1234 of 2021 on Veterinary Medicinal Products; Regulation (EU) 2019/6 |
| **6** | Is there a reference to the need for coordinating the authorization or registration of veterinary AMs with the authorization of human AMs contained in the legislation? | No legislation available |  | Yes | Veterinary medicinal products and repeal of Directive 2001/82/EC (VMP) (EU Regulation 2019/6 of 11 December 2018) (consolidated version of 28 January 2022); Veterinarians' use, dispensing and prescribing, etc. of medicinal products for animals (Veterinary Executive Order) (BEK 646 of 31 May 2023);  Amendment Order to the Veterinary Executive Order (BEK 1149 of 4 September 2023);  Guidance on the Veterinary Medicinal Products Regulation and the Veterinary Executive Order.pdf  Act on Medicinal Products (Lægemiddelloven, LBK nr 99 af 16/01/2024); Executive Order No. 1234 of 2021 on Veterinary Medicinal Products; Regulation (EU) 2019/6 |
| **7** | Does the legislation restrict the use or authorization of AMs critically important for human health which are used in animals? | No legislation available |  | Yes | Veterinary medicinal products and repeal of Directive 2001/82/EC (VMP) (EU Regulation 2019/6 of 11 December 2018) (consolidated version of 28 January 2022); Veterinarians' use, dispensing and prescribing, etc. of medicinal products for animals (Veterinary Executive Order) (BEK 646 of 31 May 2023);  Amendment Order to the Veterinary Executive Order (BEK 1149 of 4 September 2023);  Guidance on the Veterinary Medicinal Products Regulation and the Veterinary Executive Order.pdf  Act on Medicinal Products (Lægemiddelloven, LBK nr 99 af 16/01/2024); Executive Order No. 1234 of 2021 on Veterinary Medicinal Products; Regulation (EU) 2019/6Act on Medicinal Products (Lægemiddelloven, LBK nr 99 af 16/01/2024); Executive Order No. 1234 of 2021 on Veterinary Medicinal Products; Regulation (EU) 2019/6 |
| **8** | Does the law require the authorization of AMs in terms of efficacy tests, data requirement, indications/usage and claims of the product, packaging, and labelling, withdrawal periods of the drugs, acceptable daily intake (ADI), product stability when mixed with drinking water or feed, and product safety (which includes its possible effects on the human intestinal flora)? | Yes | Veterinary Surgeons and Veterinary Paraprofessionals Act, 2015 (Veterinary Medicines Directorate Regulations) | Yes | Veterinary medicinal products and repeal of Directive 2001/82/EC (VMP) (EU Regulation 2019/6 of 11 December 2018) (consolidated version of 28 January 2022); Veterinarians' use, dispensing and prescribing, etc. of medicinal products for animals (Veterinary Executive Order) (BEK 646 of 31 May 2023);  Amendment Order to the Veterinary Executive Order (BEK 1149 of 4 September 2023);  Guidance on the Veterinary Medicinal Products Regulation and the Veterinary Executive Order.pdf  Act on Medicinal Products (Lægemiddelloven, LBK nr 99 af 16/01/2024); Executive Order No. 1234 of 2021 on Veterinary Medicinal Products; Regulation (EU) 2019/6 |
| **9** | Can these requirements included in the secondary legislation be modified easily by the national authority to adapt to scientific changes? | No legislation available |  | Yes | Veterinary medicinal products and repeal of Directive 2001/82/EC (VMP) (EU Regulation 2019/6 of 11 December 2018) (consolidated version of 28 January 2022); Veterinarians' use, dispensing and prescribing, etc. of medicinal products for animals (Veterinary Executive Order) (BEK 646 of 31 May 2023);  Amendment Order to the Veterinary Executive Order (BEK 1149 of 4 September 2023);  Guidance on the Veterinary Medicinal Products Regulation and the Veterinary Executive Order.pdf  Act on Medicinal Products (Lægemiddelloven, LBK nr 99 af 16/01/2024); Executive Order No. 1234 of 2021 on Veterinary Medicinal Products; Regulation (EU) 2019/6 |
| **10** | Does the legislation require that VMPs be classified based on prescription and supply, potential hazards, and typology requirements? | Yes | Veterinary Surgeons and Veterinary Paraprofessionals Act, 2015 (Veterinary Medicines Directorate Regulations) | Yes | Veterinary medicinal products and repeal of Directive 2001/82/EC (VMP) (EU Regulation 2019/6 of 11 December 2018) (consolidated version of 28 January 2022); Veterinarians' use, dispensing and prescribing, etc. of medicinal products for animals (Veterinary Executive Order) (BEK 646 of 31 May 2023);  Amendment Order to the Veterinary Executive Order (BEK 1149 of 4 September 2023);  Guidance on the Veterinary Medicinal Products Regulation and the Veterinary Executive Order.pdf  Act on Medicinal Products (Lægemiddelloven, LBK nr 99 af 16/01/2024); Executive Order No. 1234 of 2021 on Veterinary Medicinal Products; Regulation (EU) 2019/6 |
|  |  |  |  |  |  |
| **D** | **List of essential medicines** |  |  |  |  |
| **11** | Does the legislation include the provision for approval of the list of essential medicines including veterinary medicine products [this list is distinct from the general list or registry of authorized VMPs]. | No legislation available |  | Yes |  |
| **12** | Is there a provision in legislation to restrict certain essential AMs for veterinary purposes only as the last option and in single treatments? | No legislation available |  | Yes |  |
|  |  |  |  |  |  |
| **E** | **Quality control** |  |  |  |  |
| **13** | Is there legislation that regulates the efficacy, safety, and quality of veterinary medicinal products [through the setting of product standards, control (sampling and testing), and monitoring] before registration? | Yes | Veterinary Surgeons and Veterinary Paraprofessionals Act, 2015 (Veterinary Medicines Directorate Regulations) | Yes | Veterinary medicinal products and repeal of Directive 2001/82/EC (VMP) (EU Regulation 2019/6 of 11 December 2018) (consolidated version of 28 January 2022) |
| **14** | Is there a mandate to the regulatory body to carry out pharmacovigilance (monitoring and control of veterinary medicinal products after registration and in the market) involving surveillance for side effects, and arrangements for recall? | Yes | Veterinary Surgeons and Veterinary Paraprofessionals Act, 2015 (Veterinary Medicines Directorate Regulations) | Yes | Veterinary medicinal products and repeal of Directive 2001/82/EC (VMP) (EU Regulation 2019/6 of 11 December 2018) (consolidated version of 28 January 2022) |
| **15** | Does legislation include specific provisions for the prevention, monitoring, and control of counterfeit or low-quality VMPs? | Yes | Veterinary Surgeons and Veterinary Paraprofessionals Act, 2015 (Veterinary Medicines Directorate Regulations) | Yes | Veterinary medicinal products and repeal of Directive 2001/82/EC (VMP) (EU Regulation 2019/6 of 11 December 2018) (consolidated version of 28 January 2022) |
| **16** | Are there veterinary regulatory bodies mandated with enforcement of legislation on counterfeit or low-quality VMPs? | Yes | Veterinary Surgeons and Veterinary Paraprofessionals Act, 2015 (Veterinary Medicines Directorate Regulations) | Yes | Veterinary medicinal products and repeal of Directive 2001/82/EC (VMP) (EU Regulation 2019/6 of 11 December 2018) (consolidated version of 28 January 2022) |
| **17** | Is there legislation that provides for the designation of private or public laboratories outside or within the country to test for efficacy and quality of VMPs to in order pinpoint low-quality or counterfeit veterinary drugs? | Yes | Veterinary Surgeons and Veterinary Paraprofessionals Act, 2015 (Veterinary Medicines Directorate Regulations) | Yes | Veterinary medicinal products and repeal of Directive 2001/82/EC (VMP) (EU Regulation 2019/6 of 11 December 2018) (consolidated version of 28 January 2022) |
|  |  |  |  |  |  |
| **F** | **Packages, labels, and advertisements** |  |  |  |  |
|  | Is there legislation on the labelling of antimicrobials specifying: |  |  |  |  |
| **18** | Their approved uses (which includes the animal species for which they are approved as well as the approved administration route)? | Yes | Veterinary Surgeons and Veterinary Paraprofessionals Act, 2015 (Veterinary Medicines Directorate Regulations) | Yes |  |
| **19** | approved dosages? | Yes | Veterinary Surgeons and Veterinary Paraprofessionals Act, 2015 (Veterinary Medicines Directorate Regulations) | Yes |  |
| **20** | requirements for storage? | Yes | Veterinary Surgeons and Veterinary Paraprofessionals Act, 2015 (Veterinary Medicines Directorate Regulations) | Yes |  |
| **21** | periods of drug withdrawal? | Yes | Veterinary Surgeons and Veterinary Paraprofessionals Act, 2015 (Veterinary Medicines Directorate Regulations) | Yes |  |
| **22** | Does the legislation include provisions on the incorporation of claims (for example the health claims) on the VMP label? | Yes | Veterinary Surgeons and Veterinary Paraprofessionals Act, 2015 (Veterinary Medicines Directorate Regulations) | Yes |  |
| **23** | Is there legislation on the marketing and/or advertisement of veterinary medicinal products? | Yes | Veterinary Surgeons and Veterinary Paraprofessionals Act, 2015 (Veterinary Medicines Directorate Regulations) | Yes |  |
| **24** | Are deceptive or invalid claims forbidden? | Yes | Veterinary Surgeons and Veterinary Paraprofessionals Act, 2015 (Veterinary Medicines Directorate Regulations) | Yes |  |
| **25** | Are restricted use veterinary medicinal products strictly advertised to qualified veterinary persons only and not to the public? | Yes | Veterinary Surgeons and Veterinary Paraprofessionals Act, 2015 (Veterinary Medicines Directorate Regulations) | Yes |  |
| **26** | Is it a requirement by the law that the expiry date be included on the product label? | Yes | Veterinary Surgeons and Veterinary Paraprofessionals Act, 2015 (Veterinary Medicines Directorate Regulations) | Yes |  |
| **27** | Are labels required by the law to be printed in local dialects? | No, only English | Veterinary Surgeons and Veterinary Paraprofessionals Act, 2015 (Veterinary Medicines Directorate Regulations) | Yes |  |
| **28** | Are the packages or labels required by the law to specify that the veterinary medicinal product is for veterinary use only? | Yes | Veterinary Surgeons and Veterinary Paraprofessionals Act, 2015 (Veterinary Medicines Directorate Regulations) | Yes |  |
|  |  |  |  |  |  |
| **G** | **Prescription** |  |  |  |  |
| **29** | Does the legislation prohibit the dispensing or sale of antimicrobials unprescribed? | Yes | Veterinary Surgeons and Veterinary Paraprofessionals Act, 2015 (Veterinary Medicines Directorate Regulations) | Yes | Veterinary medicinal products and repeal of Directive 2001/82/EC (VMP) (EU Regulation 2019/6 of 11 December 2018) (consolidated version of 28 January 2022); Veterinarians' use, dispensing and prescribing, etc. of medicinal products for animals (Veterinary Executive Order) (BEK 646 of 31 May 2023);  Amendment Order to the Veterinary Executive Order (BEK 1149 of 4 September 2023);  Guidance on the Veterinary Medicinal Products Regulation and the Veterinary Executive Order.pdf  Act on Medicinal Products (Lægemiddelloven, LBK nr 99 af 16/01/2024); Executive Order No. 1234 of 2021 on Veterinary Medicinal Products; Regulation (EU) 2019/6 |
| **30** | Is it restricted in the legislation that antimicrobials can be prescribed by qualified veterinary professionals only? | Yes | Veterinary Surgeons and Veterinary Paraprofessionals Act, 2015 (Veterinary Medicines Directorate Regulations) | Yes | Veterinary medicinal products and repeal of Directive 2001/82/EC (VMP) (EU Regulation 2019/6 of 11 December 2018) (consolidated version of 28 January 2022); Veterinarians' use, dispensing and prescribing, etc. of medicinal products for animals (Veterinary Executive Order) (BEK 646 of 31 May 2023);  Amendment Order to the Veterinary Executive Order (BEK 1149 of 4 September 2023);  Guidance on the Veterinary Medicinal Products Regulation and the Veterinary Executive Order.pdf  Act on Medicinal Products (Lægemiddelloven, LBK nr 99 af 16/01/2024); Executive Order No. 1234 of 2021 on Veterinary Medicinal Products; Regulation (EU) 2019/6 |
| **31** | Does the law require that qualified veterinary professionals only prescribe veterinary drugs to animals under their care depending on the clinical diagnoses? | Yes | Veterinary Surgeons and Veterinary Paraprofessionals Act, 2015 (Veterinary Medicines Directorate Regulations) | Yes | Veterinary medicinal products and repeal of Directive 2001/82/EC (VMP) (EU Regulation 2019/6 of 11 December 2018) (consolidated version of 28 January 2022); Veterinarians' use, dispensing and prescribing, etc. of medicinal products for animals (Veterinary Executive Order) (BEK 646 of 31 May 2023);  Amendment Order to the Veterinary Executive Order (BEK 1149 of 4 September 2023);  Guidance on the Veterinary Medicinal Products Regulation and the Veterinary Executive Order.pdf  Act on Medicinal Products (Lægemiddelloven, LBK nr 99 af 16/01/2024); Executive Order No. 1234 of 2021 on Veterinary Medicinal Products; Regulation (EU) 2019/6 |
| **32** | Does the law hold qualified animal health professionals accountable and responsible for how they prescribe and use the antimicrobials? | Yes | Veterinary Surgeons and Veterinary Paraprofessionals Act, 2015 (Veterinary Medicines Directorate Regulations) | Yes | Veterinary medicinal products and repeal of Directive 2001/82/EC (VMP) (EU Regulation 2019/6 of 11 December 2018) (consolidated version of 28 January 2022); Veterinarians' use, dispensing and prescribing, etc. of medicinal products for animals (Veterinary Executive Order) (BEK 646 of 31 May 2023);  Amendment Order to the Veterinary Executive Order (BEK 1149 of 4 September 2023);  Guidance on the Veterinary Medicinal Products Regulation and the Veterinary Executive Order.pdf  Act on Medicinal Products (Lægemiddelloven, LBK nr 99 af 16/01/2024); Executive Order No. 1234 of 2021 on Veterinary Medicinal Products; Regulation (EU) 2019/6 |
| **33** | Does the law require qualified animal health professionals to keep the records and share the reports on veterinary antimicrobial prescriptions? | Yes | Veterinary Surgeons and Veterinary Paraprofessionals Act, 2015 (Veterinary Medicines Directorate Regulations) | Yes | Veterinary medicinal products and repeal of Directive 2001/82/EC (VMP) (EU Regulation 2019/6 of 11 December 2018) (consolidated version of 28 January 2022); Veterinarians' use, dispensing and prescribing, etc. of medicinal products for animals (Veterinary Executive Order) (BEK 646 of 31 May 2023);  Amendment Order to the Veterinary Executive Order (BEK 1149 of 4 September 2023);  Guidance on the Veterinary Medicinal Products Regulation and the Veterinary Executive Order.pdf  Act on Medicinal Products (Lægemiddelloven, LBK nr 99 af 16/01/2024); Executive Order No. 1234 of 2021 on Veterinary Medicinal Products; Regulation (EU) 2019/6 |
| **34** | Is the veterinary regulatory body mandated to request the information on prescription of antimicrobials from all private and public veterinary professionals? | Yes | Veterinary Surgeons and Veterinary Paraprofessionals Act, 2015 (Veterinary Medicines Directorate Regulations) | Yes | Veterinary medicinal products and repeal of Directive 2001/82/EC (VMP) (EU Regulation 2019/6 of 11 December 2018) (consolidated version of 28 January 2022); Veterinarians' use, dispensing and prescribing, etc. of medicinal products for animals (Veterinary Executive Order) (BEK 646 of 31 May 2023);  Amendment Order to the Veterinary Executive Order (BEK 1149 of 4 September 2023);  Guidance on the Veterinary Medicinal Products Regulation and the Veterinary Executive Order.pdf  Act on Medicinal Products (Lægemiddelloven, LBK nr 99 af 16/01/2024); Executive Order No. 1234 of 2021 on Veterinary Medicinal Products; Regulation (EU) 2019/6 |
| **35** | Does the law include the provisions on the privacy of such records and how this data is used by the regulatory authority? | Yes | Veterinary Surgeons and Veterinary Paraprofessionals Act, 2015 (Veterinary Medicines Directorate Regulations) | Yes | Veterinary medicinal products and repeal of Directive 2001/82/EC (VMP) (EU Regulation 2019/6 of 11 December 2018) (consolidated version of 28 January 2022); Veterinarians' use, dispensing and prescribing, etc. of medicinal products for animals (Veterinary Executive Order) (BEK 646 of 31 May 2023);  Amendment Order to the Veterinary Executive Order (BEK 1149 of 4 September 2023);  Guidance on the Veterinary Medicinal Products Regulation and the Veterinary Executive Order.pdf  Act on Medicinal Products (Lægemiddelloven, LBK nr 99 af 16/01/2024); Executive Order No. 1234 of 2021 on Veterinary Medicinal Products; Regulation (EU) 2019/6 |
| **36** | Is the extra-label or off-label use of VMPs included in the legislation? | Yes | Veterinary Surgeons and Veterinary Paraprofessionals Act, 2015 (Veterinary Medicines Directorate Regulations) | Yes | Veterinary medicinal products and repeal of Directive 2001/82/EC (VMP) (EU Regulation 2019/6 of 11 December 2018) (consolidated version of 28 January 2022); Veterinarians' use, dispensing and prescribing, etc. of medicinal products for animals (Veterinary Executive Order) (BEK 646 of 31 May 2023);  Amendment Order to the Veterinary Executive Order (BEK 1149 of 4 September 2023);  Guidance on the Veterinary Medicinal Products Regulation and the Veterinary Executive Order.pdf  Act on Medicinal Products (Lægemiddelloven, LBK nr 99 af 16/01/2024); Executive Order No. 1234 of 2021 on Veterinary Medicinal Products; Regulation (EU) 2019/6 |
| **37** | Is there legislation on the prescription of anthelmintic drugs in animals? | Yes | Veterinary Surgeons and Veterinary Paraprofessionals Act, 2015 (Veterinary Medicines Directorate Regulations) | Yes | Veterinary medicinal products and repeal of Directive 2001/82/EC (VMP) (EU Regulation 2019/6 of 11 December 2018) (consolidated version of 28 January 2022); Veterinarians' use, dispensing and prescribing, etc. of medicinal products for animals (Veterinary Executive Order) (BEK 646 of 31 May 2023);  Amendment Order to the Veterinary Executive Order (BEK 1149 of 4 September 2023);  Guidance on the Veterinary Medicinal Products Regulation and the Veterinary Executive Order.pdf  Act on Medicinal Products (Lægemiddelloven, LBK nr 99 af 16/01/2024); Executive Order No. 1234 of 2021 on Veterinary Medicinal Products; Regulation (EU) 2019/6 |
| **H** | **Sales** |  |  |  |  |
| **38** | Does the legislation permit the sale of antimicrobials in veterinary clinics and pharmacies? | Yes | Veterinary Surgeons and Veterinary Paraprofessionals Act, 2015 (Veterinary Medicines Directorate Regulations) | Yes | Veterinary medicinal products and repeal of Directive 2001/82/EC (VMP) (EU Regulation 2019/6 of 11 December 2018) (consolidated version of 28 January 2022); Veterinarians' use, dispensing and prescribing, etc. of medicinal products for animals (Veterinary Executive Order) (BEK 646 of 31 May 2023);  Amendment Order to the Veterinary Executive Order (BEK 1149 of 4 September 2023);  Guidance on the Veterinary Medicinal Products Regulation and the Veterinary Executive Order.pdf  Act on Medicinal Products (Lægemiddelloven, LBK nr 99 af 16/01/2024); Executive Order No. 1234 of 2021 on Veterinary Medicinal Products; Regulation (EU) 2019/6 |
| **39** | or in other outlets like feed distribution companies? | Yes | Veterinary Surgeons and Veterinary Paraprofessionals Act, 2015 (Veterinary Medicines Directorate Regulations) | Yes | Veterinary medicinal products and repeal of Directive 2001/82/EC (VMP) (EU Regulation 2019/6 of 11 December 2018) (consolidated version of 28 January 2022); Veterinarians' use, dispensing and prescribing, etc. of medicinal products for animals (Veterinary Executive Order) (BEK 646 of 31 May 2023);  Amendment Order to the Veterinary Executive Order (BEK 1149 of 4 September 2023);  Guidance on the Veterinary Medicinal Products Regulation and the Veterinary Executive Order.pdf  Act on Medicinal Products (Lægemiddelloven, LBK nr 99 af 16/01/2024); Executive Order No. 1234 of 2021 on Veterinary Medicinal Products; Regulation (EU) 2019/6 |
| **40** | Or pet shops or supermarkets? | Yes | Veterinary Surgeons and Veterinary Paraprofessionals Act, 2015 (Veterinary Medicines Directorate Regulations) | Not allowed | Veterinary medicinal products and repeal of Directive 2001/82/EC (VMP) (EU Regulation 2019/6 of 11 December 2018) (consolidated version of 28 January 2022); Veterinarians' use, dispensing and prescribing, etc. of medicinal products for animals (Veterinary Executive Order) (BEK 646 of 31 May 2023);  Amendment Order to the Veterinary Executive Order (BEK 1149 of 4 September 2023);  Guidance on the Veterinary Medicinal Products Regulation and the Veterinary Executive Order.pdf  Act on Medicinal Products (Lægemiddelloven, LBK nr 99 af 16/01/2024); Executive Order No. 1234 of 2021 on Veterinary Medicinal Products; Regulation (EU) 2019/6 |
| **41** | Are the antimicrobials sold by different sellers differentiated in the law? | Yes | Veterinary Surgeons and Veterinary Paraprofessionals Act, 2015 (Veterinary Medicines Directorate Regulations) | Yes | Veterinary medicinal products and repeal of Directive 2001/82/EC (VMP) (EU Regulation 2019/6 of 11 December 2018) (consolidated version of 28 January 2022); Veterinarians' use, dispensing and prescribing, etc. of medicinal products for animals (Veterinary Executive Order) (BEK 646 of 31 May 2023);  Amendment Order to the Veterinary Executive Order (BEK 1149 of 4 September 2023);  Guidance on the Veterinary Medicinal Products Regulation and the Veterinary Executive Order.pdf  Act on Medicinal Products (Lægemiddelloven, LBK nr 99 af 16/01/2024); Executive Order No. 1234 of 2021 on Veterinary Medicinal Products; Regulation (EU) 2019/6 |
| **42** | Are the sellers of antimicrobials required to have specific permits or licenses? | Yes | Veterinary Surgeons and Veterinary Paraprofessionals Act, 2015 (Veterinary Medicines Directorate Regulations) | Yes | Veterinary medicinal products and repeal of Directive 2001/82/EC (VMP) (EU Regulation 2019/6 of 11 December 2018) (consolidated version of 28 January 2022); Veterinarians' use, dispensing and prescribing, etc. of medicinal products for animals (Veterinary Executive Order) (BEK 646 of 31 May 2023);  Amendment Order to the Veterinary Executive Order (BEK 1149 of 4 September 2023);  Guidance on the Veterinary Medicinal Products Regulation and the Veterinary Executive Order.pdf  Act on Medicinal Products (Lægemiddelloven, LBK nr 99 af 16/01/2024); Executive Order No. 1234 of 2021 on Veterinary Medicinal Products; Regulation (EU) 2019/6 |
| **43** | Are veterinary professionals allowed to sell antimicrobials directly to farmers? | Yes, if they have a business license | Veterinary Surgeons and Veterinary Paraprofessionals Act, 2015 (Veterinary Medicines Directorate Regulations) | No | Veterinarians Act (Veterinary Act) (LBK no. 64 of 19 January 2024); Veterinary medicinal products and repeal of Directive 2001/82/EC (VMP) (EU Regulation 2019/6 of 11 December 2018) (consolidated version of 28 January 2022); Veterinarians' use, dispensing and prescribing, etc. of medicinal products for animals (Veterinary Executive Order) (BEK 646 of 31 May 2023);  Amendment Order to the Veterinary Executive Order (BEK 1149 of 4 September 2023);  Guidance on the Veterinary Medicinal Products Regulation and the Veterinary Executive Order.pdf  Act on Medicinal Products (Lægemiddelloven, LBK nr 99 af 16/01/2024); Executive Order No. 1234 of 2021 on Veterinary Medicinal Products; Regulation (EU) 2019/6 |
| **44** | Is this activity under control? | Yes | Veterinary Surgeons and Veterinary Para-Professional Act, Cap.366, 2012) | Yes | Veterinary medicinal products and repeal of Directive 2001/82/EC (VMP) (EU Regulation 2019/6 of 11 December 2018) (consolidated version of 28 January 2022); Veterinarians' use, dispensing and prescribing, etc. of medicinal products for animals (Veterinary Executive Order) (BEK 646 of 31 May 2023);  Amendment Order to the Veterinary Executive Order (BEK 1149 of 4 September 2023);  Guidance on the Veterinary Medicinal Products Regulation and the Veterinary Executive Order.pdf  Act on Medicinal Products (Lægemiddelloven, LBK nr 99 af 16/01/2024); Executive Order No. 1234 of 2021 on Veterinary Medicinal Products; Regulation (EU) 2019/6 |
| **45** | If it is controlled, are there procedures for safeguarding against future personal interest? | Yes | Veterinary Surgeons and Veterinary Paraprofessionals Act, 2015 (Veterinary Medicines Directorate Regulations) | Yes | Veterinary medicinal products and repeal of Directive 2001/82/EC (VMP) (EU Regulation 2019/6 of 11 December 2018) (consolidated version of 28 January 2022); Veterinarians' use, dispensing and prescribing, etc. of medicinal products for animals (Veterinary Executive Order) (BEK 646 of 31 May 2023);  Amendment Order to the Veterinary Executive Order (BEK 1149 of 4 September 2023);  Guidance on the Veterinary Medicinal Products Regulation and the Veterinary Executive Order.pdf  Act on Medicinal Products (Lægemiddelloven, LBK nr 99 af 16/01/2024); Executive Order No. 1234 of 2021 on Veterinary Medicinal Products; Regulation (EU) 2019/6 |
| **46** | Are veterinarians or drug outlets such as pharmacies that trade antimicrobials required by the law to keep and report the records of antimicrobials sold? | Yes | Veterinary Surgeons and Veterinary Paraprofessionals Act, 2015 (Veterinary Medicines Directorate Regulations) | Yes | Veterinary medicinal products and repeal of Directive 2001/82/EC (VMP) (EU Regulation 2019/6 of 11 December 2018) (consolidated version of 28 January 2022); Veterinarians' use, dispensing and prescribing, etc. of medicinal products for animals (Veterinary Executive Order) (BEK 646 of 31 May 2023);  Amendment Order to the Veterinary Executive Order (BEK 1149 of 4 September 2023);  Guidance on the Veterinary Medicinal Products Regulation and the Veterinary Executive Order.pdf  Act on Medicinal Products (Lægemiddelloven, LBK nr 99 af 16/01/2024); Executive Order No. 1234 of 2021 on Veterinary Medicinal Products; Regulation (EU) 2019/6 |
| **47** | Is the information to be recorded clearly stated in the law? | Yes | Veterinary Surgeons and Veterinary Paraprofessionals Act, 2015 (Veterinary Medicines Directorate Regulations) | Yes | Veterinarians Act (Veterinary Act) (LBK no. 64 of 19 January 2024); Veterinary medicinal products and repeal of Directive 2001/82/EC (VMP) (EU Regulation 2019/6 of 11 December 2018) (consolidated version of 28 January 2022); Veterinarians' use, dispensing and prescribing, etc. of medicinal products for animals (Veterinary Executive Order) (BEK 646 of 31 May 2023);  Amendment Order to the Veterinary Executive Order (BEK 1149 of 4 September 2023);  Guidance on the Veterinary Medicinal Products Regulation and the Veterinary Executive Order.pdf  Act on Medicinal Products (Lægemiddelloven, LBK nr 99 af 16/01/2024); Executive Order No. 1234 of 2021 on Veterinary Medicinal Products; Regulation (EU) 2019/6 |
| **48** | Are there any restrictions on whom AMs can be sold to? | Yes | Veterinary Surgeons and Veterinary Paraprofessionals Act, 2015 (Veterinary Medicines Directorate Regulations) | Yes | Veterinarians Act (Veterinary Act) (LBK no. 64 of 19 January 2024); Veterinary medicinal products and repeal of Directive 2001/82/EC (VMP) (EU Regulation 2019/6 of 11 December 2018) (consolidated version of 28 January 2022); Veterinarians' use, dispensing and prescribing, etc. of medicinal products for animals (Veterinary Executive Order) (BEK 646 of 31 May 2023);  Amendment Order to the Veterinary Executive Order (BEK 1149 of 4 September 2023);  Guidance on the Veterinary Medicinal Products Regulation and the Veterinary Executive Order.pdf  Act on Medicinal Products (Lægemiddelloven, LBK nr 99 af 16/01/2024); Executive Order No. 1234 of 2021 on Veterinary Medicinal Products; Regulation (EU) 2019/6 |
| **49** | Is there a restriction on counterfeit, poor-quality, unregistered or unlabelled antimicrobials? | Yes | Veterinary Surgeons and Veterinary Paraprofessionals Act, 2015 (Veterinary Medicines Directorate Regulations) | Yes | Veterinarians Act (Veterinary Act) (LBK no. 64 of 19 January 2024); Veterinary medicinal products and repeal of Directive 2001/82/EC (VMP) (EU Regulation 2019/6 of 11 December 2018) (consolidated version of 28 January 2022); Veterinarians' use, dispensing and prescribing, etc. of medicinal products for animals (Veterinary Executive Order) (BEK 646 of 31 May 2023);  Amendment Order to the Veterinary Executive Order (BEK 1149 of 4 September 2023);  Guidance on the Veterinary Medicinal Products Regulation and the Veterinary Executive Order.pdf  Act on Medicinal Products (Lægemiddelloven, LBK nr 99 af 16/01/2024); Executive Order No. 1234 of 2021 on Veterinary Medicinal Products; Regulation (EU) 2019/6 |
| **50** | Are the preferred storage conditions of antimicrobials specified in the legislation? | Yes | Veterinary Surgeons and Veterinary Paraprofessionals Act, 2015 (Veterinary Medicines Directorate Regulations) | Yes | Veterinarians Act (Veterinary Act) (LBK no. 64 of 19 January 2024); Veterinary medicinal products and repeal of Directive 2001/82/EC (VMP) (EU Regulation 2019/6 of 11 December 2018) (consolidated version of 28 January 2022); Veterinarians' use, dispensing and prescribing, etc. of medicinal products for animals (Veterinary Executive Order) (BEK 646 of 31 May 2023);  Amendment Order to the Veterinary Executive Order (BEK 1149 of 4 September 2023);  Guidance on the Veterinary Medicinal Products Regulation and the Veterinary Executive Order.pdf  Act on Medicinal Products (Lægemiddelloven, LBK nr 99 af 16/01/2024); Executive Order No. 1234 of 2021 on Veterinary Medicinal Products; Regulation (EU) 2019/6 |
| **51** | Are there requirements for the disposal of unused and expired AMs? | Yes | Veterinary Surgeons and Veterinary Paraprofessionals Act, 2015 (Veterinary Medicines Directorate Regulations) | Yes | Veterinarians Act (Veterinary Act) (LBK no. 64 of 19 January 2024); Veterinary medicinal products and repeal of Directive 2001/82/EC (VMP) (EU Regulation 2019/6 of 11 December 2018) (consolidated version of 28 January 2022); Veterinarians' use, dispensing and prescribing, etc. of medicinal products for animals (Veterinary Executive Order) (BEK 646 of 31 May 2023);  Amendment Order to the Veterinary Executive Order (BEK 1149 of 4 September 2023);  Guidance on the Veterinary Medicinal Products Regulation and the Veterinary Executive Order.pdf  Act on Medicinal Products (Lægemiddelloven, LBK nr 99 af 16/01/2024); Executive Order No. 1234 of 2021 on Veterinary Medicinal Products; Regulation (EU) 2019/6 |
| **52** | Does the legislation contain a provision on penalties for the persons or drug outlets that sell antimicrobials in a manner contrary to the legislation? | Yes | Veterinary Surgeons and Veterinary Paraprofessionals Act, 2015 (Veterinary Medicines Directorate Regulations) | Yes | Veterinarians Act (Veterinary Act) (LBK no. 64 of 19 January 2024); Veterinary medicinal products and repeal of Directive 2001/82/EC (VMP) (EU Regulation 2019/6 of 11 December 2018) (consolidated version of 28 January 2022); Veterinarians' use, dispensing and prescribing, etc. of medicinal products for animals (Veterinary Executive Order) (BEK 646 of 31 May 2023);  Amendment Order to the Veterinary Executive Order (BEK 1149 of 4 September 2023);  Guidance on the Veterinary Medicinal Products Regulation and the Veterinary Executive Order.pdf  Act on Medicinal Products (Lægemiddelloven, LBK nr 99 af 16/01/2024); Executive Order No. 1234 of 2021 on Veterinary Medicinal Products; Regulation (EU) 2019/6 |
| **I** | **Use** |  |  |  |  |
| **54** | Is there a provision with the rules on ways of selling the antimicrobials? | Yes | Veterinary Surgeons and Veterinary Paraprofessionals Act, 2015 (Veterinary Medicines Directorate Regulations) | Yes | Veterinarians' use, dispensing and prescribing, etc. of medicinal products for animals (Veterinary Executive Order) (BEK 646 of 31 May 2023);  Amendment Order to the Veterinary Executive Order (BEK 1149 of 4 September 2023);  Guidance on the Veterinary Medicinal Products Regulation and the Veterinary Executive Order. |
| **55** | Is the supervision of antimicrobial use or administration by veterinary professionals under restrictions? | Yes | Veterinary Surgeons and Veterinary Para-Professional Act, Cap.366, 2012) | Yes | Veterinarians' use, dispensing and prescribing, etc. of medicinal products for animals (Veterinary Executive Order) (BEK 646 of 31 May 2023);  Amendment Order to the Veterinary Executive Order (BEK 1149 of 4 September 2023);  Guidance on the Veterinary Medicinal Products Regulation and the Veterinary Executive Order. |
| **56** | Does the legislation differentiate between non-therapeutic and therapeutic antimicrobial uses? | No legislation available |  | Yes | Veterinarians' use, dispensing and prescribing, etc. of medicinal products for animals (Veterinary Executive Order) (BEK 646 of 31 May 2023);  Amendment Order to the Veterinary Executive Order (BEK 1149 of 4 September 2023);  Guidance on the Veterinary Medicinal Products Regulation and the Veterinary Executive Order. |
| **57** | Are the non-therapeutic antimicrobial uses, especially for growth promotion, under restrictions? | No legislation available |  | Yes | Veterinarians' use, dispensing and prescribing, etc. of medicinal products for animals (Veterinary Executive Order) (BEK 646 of 31 May 2023);  Amendment Order to the Veterinary Executive Order (BEK 1149 of 4 September 2023);  Guidance on the Veterinary Medicinal Products Regulation and the Veterinary Executive Order. |
| **58** | Are there risk criteria for disease diagnosis for preventive purposes in veterinary metaphylaxis and prophylaxis? | No legislation available |  | Yes | Veterinarians' use, dispensing and prescribing, etc. of medicinal products for animals (Veterinary Executive Order) (BEK 646 of 31 May 2023);  Amendment Order to the Veterinary Executive Order (BEK 1149 of 4 September 2023);  Guidance on the Veterinary Medicinal Products Regulation and the Veterinary Executive Order. |
| **59** | Is the use of human medically important antimicrobials in veterinary animal species under any restrictions? | No legislation available |  | Yes | Veterinarians' use, dispensing and prescribing, etc. of medicinal products for animals (Veterinary Executive Order) (BEK 646 of 31 May 2023);  Amendment Order to the Veterinary Executive Order (BEK 1149 of 4 September 2023);  Guidance on the Veterinary Medicinal Products Regulation and the Veterinary Executive Order. |
| **60** | Does the law define the terms treatment, control, and prevention? | Yes | Veterinary Surgeons and Veterinary Para-Professional Act, Cap.366, 2012) | Yes | Veterinarians' use, dispensing and prescribing, etc. of medicinal products for animals (Veterinary Executive Order) (BEK 646 of 31 May 2023);  Amendment Order to the Veterinary Executive Order (BEK 1149 of 4 September 2023);  Guidance on the Veterinary Medicinal Products Regulation and the Veterinary Executive Order. |
| **61** | Are the therapeutic uses of antimicrobials restricted? | Yes | Veterinary Surgeons and Veterinary Paraprofessionals Act, 2015 (Veterinary Medicines Directorate Regulations) | Yes | Veterinarians' use, dispensing and prescribing, etc. of medicinal products for animals (Veterinary Executive Order) (BEK 646 of 31 May 2023);  Amendment Order to the Veterinary Executive Order (BEK 1149 of 4 September 2023);  Guidance on the Veterinary Medicinal Products Regulation and the Veterinary Executive Order. |
| **62** | Are these restrictions dependent on the analysis of risks? | Yes | Veterinary Surgeons and Veterinary Paraprofessionals Act, 2015 (Veterinary Medicines Directorate Regulations) | Yes | Veterinarians' use, dispensing and prescribing, etc. of medicinal products for animals (Veterinary Executive Order) (BEK 646 of 31 May 2023);  Amendment Order to the Veterinary Executive Order (BEK 1149 of 4 September 2023);  Guidance on the Veterinary Medicinal Products Regulation and the Veterinary Executive Order. |
| **63** | Is it a requirement that veterinarians or other authorized professionals administer antimicrobials into animals or oversee this activity? | Yes | Veterinary Surgeons and Veterinary Paraprofessionals Act, 2015 (Veterinary Medicines Directorate Regulations) | Yes | Veterinarians' use, dispensing and prescribing, etc. of medicinal products for animals (Veterinary Executive Order) (BEK 646 of 31 May 2023);  Amendment Order to the Veterinary Executive Order (BEK 1149 of 4 September 2023);  Guidance on the Veterinary Medicinal Products Regulation and the Veterinary Executive Order. |
| **64** | Are the aquaculture or livestock farmers required by the law to record the antimicrobials they use and to report them to the relevant national authority? | Yes | Guidelines for prudent use of antimicrobials in animals | Yes | Animal owners' use of medicines for animals, etc. (Animal Owners' Executive Order) (BEK 645 of 31 May 2023).  Guidance on the Veterinary Medicinal Products Regulation and the Animal Owners Executive Order. |
| **65** | Are the farmers allowed to take the samples for laboratory testing? | Yes | Guidelines for prudent use of antimicrobials in animals | Yes | Animal owners' use of medicines for animals, etc. (Animal Owners' Executive Order) (BEK 645 of 31 May 2023).  Guidance on the Veterinary Medicinal Products Regulation and the Animal Owners Executive Order. |
| **66** | Does the law require that the farmers return the expired or unused antimicrobials to the point of sale? | No legislation available |  | Yes | Animal owners' use of medicines for animals, etc. (Animal Owners' Executive Order) (BEK 645 of 31 May 2023).  Guidance on the Veterinary Medicinal Products Regulation and the Animal Owners Executive Order. |
| **67** | Is it specified by the law how farmers should dispose of expired or unused antimicrobials? | Yes | Guidelines on veterinary medicinal products waste management | Yes | Animal owners' use of medicines for animals, etc. (Animal Owners' Executive Order) (BEK 645 of 31 May 2023).  Guidance on the Veterinary Medicinal Products Regulation and the Animal Owners Executive Order. |
| **68** | Does the legislation allow the prophylactic use of antimicrobials in animal production? | Yes | Veterinary Surgeons and Veterinary Paraprofessionals Act, 2015 (Veterinary Medicines Directorate Regulations) | No | Animal owners' use of medicines for animals, etc. (Animal Owners' Executive Order) (BEK 645 of 31 May 2023).  Guidance on the Veterinary Medicinal Products Regulation and the Animal Owners Executive Order. |
|  |  |  |  |  |  |
| **J** | **Manufacturing** |  |  |  |  |
| **69** | Are veterinary medicinal products manufactured in the country? | Yes (mostly repackaging of FFP) | Veterinary Surgeons and Veterinary Paraprofessionals Act, 2015 (Veterinary Medicines Directorate Regulations) | Yes | Veterinary medicinal products and repeal of Directive 2001/82/EC (VMP) (EU Regulation 2019/6 of 11 December 2018) (consolidated version of 28 January 2022);  Veterinarians' use, dispensing and prescribing, etc. of medicinal products for animals (Veterinary Executive Order) (BEK 646 of 31 May 2023);  Amendment Order to the Veterinary Executive Order (BEK 1149 of 4 September 2023);  Guidance on the Veterinary Medicinal Products Regulation and the Veterinary Executive Order. |
| **70** | Does the law contain the provision on procedures of drug manufacture and requirements for quality control? | Yes | Veterinary Surgeons and Veterinary Paraprofessionals Act, 2015 (Veterinary Medicines Directorate Regulations) | Yes | Veterinary medicinal products and repeal of Directive 2001/82/EC (VMP) (EU Regulation 2019/6 of 11 December 2018) (consolidated version of 28 January 2022);  Veterinarians' use, dispensing and prescribing, etc. of medicinal products for animals (Veterinary Executive Order) (BEK 646 of 31 May 2023);  Amendment Order to the Veterinary Executive Order (BEK 1149 of 4 September 2023);  Guidance on the Veterinary Medicinal Products Regulation and the Veterinary Executive Order. |
| **71** | Is the procedure of authorizing or registering the manufacturers of veterinary medicinal products stated in the legislation? | Yes | Veterinary Surgeons and Veterinary Paraprofessionals Act, 2015 (Veterinary Medicines Directorate Regulations) | Yes | Veterinary medicinal products and repeal of Directive 2001/82/EC (VMP) (EU Regulation 2019/6 of 11 December 2018) (consolidated version of 28 January 2022);  Veterinarians' use, dispensing and prescribing, etc. of medicinal products for animals (Veterinary Executive Order) (BEK 646 of 31 May 2023);  Amendment Order to the Veterinary Executive Order (BEK 1149 of 4 September 2023);  Guidance on the Veterinary Medicinal Products Regulation and the Veterinary Executive Order. |
| **72** | Are the manufacturers of veterinary products bound by the law to keep records of antimicrobials produced and their quantities, and report them to the regulatory authorities in the country? | Yes | Veterinary Surgeons and Veterinary Paraprofessionals Act, 2015 (Veterinary Medicines Directorate Regulations) | Yes | Veterinary medicinal products and repeal of Directive 2001/82/EC (VMP) (EU Regulation 2019/6 of 11 December 2018) (consolidated version of 28 January 2022);  Veterinarians' use, dispensing and prescribing, etc. of medicinal products for animals (Veterinary Executive Order) (BEK 646 of 31 May 2023);  Amendment Order to the Veterinary Executive Order (BEK 1149 of 4 September 2023);  Guidance on the Veterinary Medicinal Products Regulation and the Veterinary Executive Order. |
| **73** | Is it provided by the law on minimizing the contamination of the environment with the process of antimicrobial production? | Yes | Pharmacy and Poisons Act, Cap.244, 2022 | Yes | Veterinary medicinal products and repeal of Directive 2001/82/EC (VMP) (EU Regulation 2019/6 of 11 December 2018) (consolidated version of 28 January 2022);  Veterinarians' use, dispensing and prescribing, etc. of medicinal products for animals (Veterinary Executive Order) (BEK 646 of 31 May 2023);  Amendment Order to the Veterinary Executive Order (BEK 1149 of 4 September 2023);  Guidance on the Veterinary Medicinal Products Regulation and the Veterinary Executive Order. |
| **74** | Are the manufacturers restricted or prohibited from repackaging the antimicrobials? | Yes, are allowed | Veterinary Surgeons and Veterinary Paraprofessionals Act, 2015 (Veterinary Medicines Directorate Regulations) | Yes | Veterinary medicinal products and repeal of Directive 2001/82/EC (VMP) (EU Regulation 2019/6 of 11 December 2018) (consolidated version of 28 January 2022);  Veterinarians' use, dispensing and prescribing, etc. of medicinal products for animals (Veterinary Executive Order) (BEK 646 of 31 May 2023);  Amendment Order to the Veterinary Executive Order (BEK 1149 of 4 September 2023);  Guidance on the Veterinary Medicinal Products Regulation and the Veterinary Executive Order. |
| **K** | **Animal Health** |  |  |  |  |
| **75** | Does the legislation identify the relevant veterinary authority and its power to implement the legislation? | Yes | Veterinary Surgeons and Veterinary Para-Professional Act, Cap.366, 2012) | Yes | Veterinarians Act (Veterinary Act) (LBK no. 64 of 19 January 2024); Act on the Keeping of Animals (LBK no. 62 of 19 January 2024) |
| 76 | Name the relevant veterinary authority. | Directorate of Veterinary Services | Veterinary Surgeons and Veterinary Para-Professional Act, Cap.366, 2012)  The Disease Act, Cap.364, 2022 | The Danish Veterinary and Food Administration (DVFA) | Veterinarians Act (Veterinary Act) (LBK no. 64 of 19 January 2024); Act on the Keeping of Animals (LBK no. 62 of 19 January 2024) |
| 77 | Is the relevant authority empowered to control or restrict animal and animal product movement? | Yes | The Disease Act, Cap.364, 2022 | Yes | Veterinarians Act (Veterinary Act) (LBK no. 64 of 19 January 2024); |
| 78 | Is the relevant authority mandated to authorize the disease surveillance which includes the collection and analysis of the required samples? | Yes | The Disease Act, Cap.364, 2022 | Yes | Veterinarians Act (Veterinary Act) (LBK no. 64 of 19 January 2024) |
| 79 | Are the laboratories obligated to share the surveillance data with other accredited laboratories and references? | Yes | Veterinary Surgeons and Veterinary Paraprofessionals Act, 2015 (Veterinary Medicines Directorate Regulations) | Yes | Veterinarians Act (Veterinary Act) (LBK no. 64 of 19 January 2024) |
| 80 | Is the relevant authority empowered to approve other disease control measures such as quarantine, control plans, culling of unhealthy animals, mandatory vaccinations or medicaments, and indemnity? | Yes | The Disease Act, Cap.364, 2022 | Yes | Veterinarians Act (Veterinary Act) (LBK no. 64 of 19 January 2024) |
| 81 | Does legislation recognize the responsibility of producers to maintain the health status of their animals, keep records, notify potential diseases, and implement bio-security measures? | Yes | Guidelines for prudent use of antimicrobials in animals | Yes | Veterinarians Act (Veterinary Act) (LBK no. 64 of 19 January 2024) |
| 82 | Does legislation establish a system for animal identification and traceability? | Yes | Animal identification and Traceability Strategy (2020-2030) | Yes | Veterinarians Act (Veterinary Act) (LBK no. 64 of 19 January 2024) |
| 83 | Does legislation give the mandate to the relevant authority to monitor and enforce veterinary legislation, including the powers to enter private properties, take samples, and review records? | Yes | The Disease Act, Cap.364, 2022 | Yes | Veterinarians Act (Veterinary Act) (LBK no. 64 of 19 January 2024) |
| 84 | Does the relevant authority have the right to request surveillance on antimicrobial resistance? | Yes | Guidelines for prudent use of antimicrobials in animals | Yes | Veterinarians Act (Veterinary Act) (LBK no. 64 of 19 January 2024) |
| 85 | Are there specified qualifications for persons who need to prescribe the antimicrobials? | Yes | Veterinary Surgeons and Veterinary Paraprofessionals Act, 2015 (Veterinary Medicines Directorate Regulations) | Yes | Veterinarians Act (Veterinary Act) (LBK no. 64 of 19 January 2024) |
| **L** | **Animal production** |  |  |  |  |
| **86** | Is there legislation on animal welfare that includes good animal husbandry and production practices? | Yes | The Prevention of Cruelty to Animals Act, Cap.360, 2022 | Yes | Act on the Keeping of Animals (LBK no. 62 of 19 January 2024) |
| **87** | Are there protocols for veterinary health facilities and abattoirs for minimizing the spread of microorganisms or contamination with antimicrobials? | Yes | Meat Control Act, Cap. 356, 2012; The Veterinary Surgeons and Veterinary Paraprofessionals (Code of Ethics) Regulations, 2011 | Yes | Act on the Keeping of Animals (LBK no. 62 of 19 January 2024) |
| **88** | Does the legislation contain the provisions for these protocols? | Yes | Meat Control Act, Cap. 356, 2012; The Veterinary Surgeons and Veterinary Paraprofessionals (Code of Ethics) Regulations, 2011 | Yes | Act on the Keeping of Animals (LBK no. 62 of 19 January 2024) |
| **89** | Are there protocols for farms on lowering the spread of microorganisms or contaminations with antimicrobials? | Yes | Guidelines for prudent use of antimicrobials in animals | Yes | Act on the Keeping of Animals (LBK no. 62 of 19 January 2024) |
| **90** | Are farmers restricted from using manure of animals treated with antimicrobials as fertilizers? | No legislation available |  | Yes | Act on the Keeping of Animals (LBK no. 62 of 19 January 2024) |
| **91** | Are there guidelines on the disposal of antimicrobial-treated animals and their by-products, together with water and wastes that result from cleaning the premises of animals treated with AMs? | No legislation available |  | Yes | Act on the Keeping of Animals (LBK no. 62 of 19 January 2024) |
| **92** | Are there guidelines on the storage and disposal of AMs on the farm? | Yes | Guidelines for prudent use of antimicrobials in animals  Guidelines on veterinary medicinal products waste management | Yes | Act on the Keeping of Animals (LBK no. 62 of 19 January 2024) |
| **93** | Are there protocols for using antimicrobials in drinking water systems? | Yes | National guidelines for safe management of healthcare waste | Yes | Act on the Keeping of Animals (LBK no. 62 of 19 January 2024) |
| **94** | Does the law provide safety and health at the place of work for the protection of farmers from the risks associated with exposure to antimicrobials and bacteria resistant to antimicrobial therapy? | Yes | Guidelines for prudent use of antimicrobials in animals | Yes | Act on the Keeping of Animals (LBK no. 62 of 19 January 2024) |
| **M** | **Medicated feed** |  |  |  |  |
| **95** | Is there legislation on AM-medicated feed? | Yes | Veterinary Surgeons and Veterinary Paraprofessionals Act, 2015 (Veterinary Medicines Directorate Regulations)  Guidelines for prudent use of antimicrobials in animals | Yes | ​Feed and feed companies (Feed Executive Order) (BEK 2227 of 30 November 2021); Regulation 2019/4 on the manufacture, placing on the market and use of medicated feed, amending Regulation (EC) No 183/2005 of the European Parliament and of the Council and repealing Council Directive 90/167/EEC; Feed and feed companies (Feed Guide) (VEJ 9006 of 10 January 2023) |
| **96** | Are medicated feeds defined in the legislation? | Yes | Veterinary Surgeons and Veterinary Paraprofessionals Act, 2015 (Veterinary Medicines Directorate Regulations) | Yes | Feed and feed companies (Feed Executive Order) (BEK 2227 of 30 November 2021); Regulation 2019/4 on the manufacture, placing on the market and use of medicated feed, amending Regulation (EC) No 183/2005 of the European Parliament and of the Council and repealing Council Directive 90/167/EEC; Feed and feed companies (Feed Guide) (VEJ 9006 of 10 January 2023) |
| **97** | Does the definition incorporate other AMs such as anti-parasitics? | Yes | Veterinary Surgeons and Veterinary Paraprofessionals Act, 2015 (Veterinary Medicines Directorate Regulations) | Yes | Feed and feed companies (Feed Executive Order) (BEK 2227 of 30 November 2021); Regulation 2019/4 on the manufacture, placing on the market and use of medicated feed, amending Regulation (EC) No 183/2005 of the European Parliament and of the Council and repealing Council Directive 90/167/EEC; Feed and feed companies (Feed Guide) (VEJ 9006 of 10 January 2023) |
| **98** | Are antimicrobial-medicated feeds subjected to the same requirements (such as authorization, use, sale, and prescription) as conventional VMPs? | No legislation available |  | Yes | Feed and feed companies (Feed Executive Order) (BEK 2227 of 30 November 2021); Regulation 2019/4 on the manufacture, placing on the market and use of medicated feed, amending Regulation (EC) No 183/2005 of the European Parliament and of the Council and repealing Council Directive 90/167/EEC; Feed and feed companies (Feed Guide) (VEJ 9006 of 10 January 2023) |
| **99** | Are the manufacture, importation, selling, and use of antimicrobial-medicated feed restricted? | No legislation available |  | Yes | Feed and feed companies (Feed Executive Order) (BEK 2227 of 30 November 2021); Regulation 2019/4 on the manufacture, placing on the market and use of medicated feed, amending Regulation (EC) No 183/2005 of the European Parliament and of the Council and repealing Council Directive 90/167/EEC; Feed and feed companies (Feed Guide) (VEJ 9006 of 10 January 2023) |
| **100** | Are the non-therapeutic uses (such as promotion of growth) of medicated feed under prohibition or restriction? | No legislation available |  | Yes | Feed and feed companies (Feed Executive Order) (BEK 2227 of 30 November 2021); Regulation 2019/4 on the manufacture, placing on the market and use of medicated feed, amending Regulation (EC) No 183/2005 of the European Parliament and of the Council and repealing Council Directive 90/167/EEC; Feed and feed companies (Feed Guide) (VEJ 9006 of 10 January 2023) |
| **101** | Is the selling or dispensing of unprescribed medicated feed prohibited? | No legislation |  | Yes | Feed and feed companies (Feed Executive Order) (BEK 2227 of 30 November 2021); Regulation 2019/4 on the manufacture, placing on the market and use of medicated feed, amending Regulation (EC) No 183/2005 of the European Parliament and of the Council and repealing Council Directive 90/167/EEC; Feed and feed companies (Feed Guide) (VEJ 9006 of 10 January 2023) |
| **102** | Are there restrictions on the manufacture or addition of antimicrobials in the feed? | Yes | Veterinary Surgeons and Veterinary Paraprofessionals Act, 2015 (Veterinary Medicines Directorate Regulations) | Yes | Feed and feed companies (Feed Executive Order) (BEK 2227 of 30 November 2021); Regulation 2019/4 on the manufacture, placing on the market and use of medicated feed, amending Regulation (EC) No 183/2005 of the European Parliament and of the Council and repealing Council Directive 90/167/EEC; Feed and feed companies (Feed Guide) (VEJ 9006 of 10 January 2023) |
| **103** | Are there guidelines that avert the contamination of other feed products with antimicrobial-medicated feed? | Yes | Veterinary Surgeons and Veterinary Paraprofessionals Act, 2015 (Veterinary Medicines Directorate Regulations) | Yes | Feed and feed companies (Feed Executive Order) (BEK 2227 of 30 November 2021); Regulation 2019/4 on the manufacture, placing on the market and use of medicated feed, amending Regulation (EC) No 183/2005 of the European Parliament and of the Council and repealing Council Directive 90/167/EEC; Feed and feed companies (Feed Guide) (VEJ 9006 of 10 January 2023) |
| **104** | Is the prescription by the veterinarian required before the medicated feed is purchased? | Yes | Veterinary Surgeons and Veterinary Paraprofessionals Act, 2015 (Veterinary Medicines Directorate Regulations) | Yes | Feed and feed companies (Feed Executive Order) (BEK 2227 of 30 November 2021); Regulation 2019/4 on the manufacture, placing on the market and use of medicated feed, amending Regulation (EC) No 183/2005 of the European Parliament and of the Council and repealing Council Directive 90/167/EEC; Feed and feed companies (Feed Guide) (VEJ 9006 of 10 January 2023) |
| **105** | Does the legislation provide the medicated feed labelling requirements? | Yes | Veterinary Surgeons and Veterinary Paraprofessionals Act, 2015 (Veterinary Medicines Directorate Regulations) | Yes | Feed and feed companies (Feed Executive Order) (BEK 2227 of 30 November 2021); Regulation 2019/4 on the manufacture, placing on the market and use of medicated feed, amending Regulation (EC) No 183/2005 of the European Parliament and of the Council and repealing Council Directive 90/167/EEC; Feed and feed companies (Feed Guide) (VEJ 9006 of 10 January 2023) |
| **106** | Does the legislation provide on the medicated feed labelling requirements? | Yes | Veterinary Surgeons and Veterinary Paraprofessionals Act, 2015 (Veterinary Medicines Directorate Regulations) | Yes | Feed and feed companies (Feed Executive Order) (BEK 2227 of 30 November 2021); Regulation 2019/4 on the manufacture, placing on the market and use of medicated feed, amending Regulation (EC) No 183/2005 of the European Parliament and of the Council and repealing Council Directive 90/167/EEC; Feed and feed companies (Feed Guide) (VEJ 9006 of 10 January 2023) |
|  |  |  |  |  |  |
| **N** | **Animal feed supplements** |  |  |  |  |
| **107** | Are feed supplements defined in the legislation? | Yes | Veterinary Surgeons and Veterinary Para-Professional Act, Cap.366, 2012 | Yes | Feed and feed companies (Feed Executive Order) (BEK 2227 of 30 November 2021); Regulation 2019/4 on the manufacture, placing on the market and use of medicated feed, amending Regulation (EC) No 183/2005 of the European Parliament and of the Council and repealing Council Directive 90/167/EEC; Feed and feed companies (Feed Guide) (VEJ 9006 of 10 January 2023) |
| **108** | Is the relevant authority mandated with authorization, approval, prohibition, or restriction on the use of feed supplements in livestock production? | Yes | The Veterinary Surgeons and Veterinary Paraprofessionals (Code of Ethics) Regulations, 2011 | Yes | Feed and feed companies (Feed Executive Order) (BEK 2227 of 30 November 2021); Regulation 2019/4 on the manufacture, placing on the market and use of medicated feed, amending Regulation (EC) No 183/2005 of the European Parliament and of the Council and repealing Council Directive 90/167/EEC; Feed and feed companies (Feed Guide) (VEJ 9006 of 10 January 2023) |
| **109** | Does the legislation allow the “growth” or “health” claims for feed supplements which are non-veterinary medicinal products (such as probiotics) that may have positive effects on the health of animals? | Yes | The Veterinary Surgeons and Veterinary Paraprofessionals (Code of Ethics) Regulations, 2011 | Yes | Feed and feed companies (Feed Executive Order) (BEK 2227 of 30 November 2021); Regulation 2019/4 on the manufacture, placing on the market and use of medicated feed, amending Regulation (EC) No 183/2005 of the European Parliament and of the Council and repealing Council Directive 90/167/EEC; Feed and feed companies (Feed Guide) (VEJ 9006 of 10 January 2023) |
| **O** | **Food safety** |  |  |  |  |
| **110** | Are relevant national authorities mandated to authorize measures for preventing, identifying, and controlling the hazards in food hazards including contamination with microorganisms and antimicrobials (antimicrobial residues and AMR)? | Yes | Food, Drugs and Chemical Substances Act, Cap. 254, 2012; Meat Control Act, Cap. 356, 2012; The Public Health Act, Cap.242, 2022 | Yes | Food Act (No. 46 of 2017)  Food Quality Act (No. 402 of 1997) |
| **111** | Do these authorities have the mandate of approving, monitoring, and controlling the standards of food quality and safety in line with Codex standards? | Yes | Food, Drugs and Chemical Substances Act, Cap. 254, 2012; Meat Control Act, Cap. 356, 2012; The Public Health Act, Cap.242, 2022 | Yes | Food Act (No. 46 of 2017)  Food Quality Act (No. 402 of 1997)  Food Regulation: Regulation (EC) No 178/2002 of the European Parliament and of the Council of 28 January 2002 laying down the general principles and requirements of food law, establishing the European Food Safety Authority and laying down procedures in matters of food safety |
| **112** | Is the regulatory authority for food safety empowered to carry out surveillance programs for identification of food hazards (such as microorganisms, antimicrobial residues, and AMR) | Yes | Food, Drugs and Chemical Substances Act, Cap. 254, 2012; Meat Control Act, Cap. 356, 2012; The Public Health Act, Cap.242, 2022 | Yes | Food Act (No. 46 of 2017)  Food Quality Act (No. 402 of 1997)  Microbiology Regulation: Commission Regulation (EC) No 2073/2005 of 15 November 2005 on microbiological criteria for foodstuffs (; Food Regulation: Regulation (EC) No 178/2002 of the European Parliament and of the Council of 28 January 2002 laying down the general principles and requirements of food law, establishing the European Food Safety Authority and laying down procedures in matters of food safety ; Control Regulation: Regulation (EU) No 2017/625 of the European Parliament and of the Council of 15 March 2017 on official controls and other official activities performed to ensure the application of food and feed law, animal health and welfare rules, plant health and plant protection products; Commission Regulation (EC) No 2074/2005 of 5 December 2005 laying down detailed rules for the implementation of Regulation (EC) No 853/2004 of the European Parliament and of the Council as regards certain products and of official controls pursuant to Regulations (EC) No 854/2004 and (EC) No 882/2004 of the European Parliament and of the Council, derogating from Regulation (EC) No 852/2004 of the European Parliament and of the Council and amending Regulations (EC) No 853/2004 and (EC) No 854/2004 (latest version) |
| **113** | Is the provision on traceability and recall of food included in the legislation? | Yes | Food, Drugs and Chemical Substances Act, Cap. 254, 2012; Meat Control Act, Cap. 356, 2012; The Public Health Act, Cap.242, 2022 | Yes | Food Act (No. 46 of 2017)  Food Quality Act (No. 402 of 1997)  Microbiology Regulation: Commission Regulation (EC) No 2073/2005 of 15 November 2005 on microbiological criteria for foodstuffs (; Food Regulation: Regulation (EC) No 178/2002 of the European Parliament and of the Council of 28 January 2002 laying down the general principles and requirements of food law, establishing the European Food Safety Authority and laying down procedures in matters of food safety ; Control Regulation: Regulation (EU) No 2017/625 of the European Parliament and of the Council of 15 March 2017 on official controls and other official activities performed to ensure the application of food and feed law, animal health and welfare rules, plant health and plant protection products; Commission Regulation (EC) No 2074/2005 of 5 December 2005 laying down detailed rules for the implementation of Regulation (EC) No 853/2004 of the European Parliament and of the Council as regards certain products and of official controls pursuant to Regulations (EC) No 854/2004 and (EC) No 882/2004 of the European Parliament and of the Council, derogating from Regulation (EC) No 852/2004 of the European Parliament and of the Council and amending Regulations (EC) No 853/2004 and (EC) No 854/2004 (latest version) |
| **114** | Is there a mechanism for declaring a food-borne outbreak and containing the risk based on the measures of food safety? | Yes | Food, Drugs and Chemical Substances Act, Cap. 254, 2012; Meat Control Act, Cap. 356, 2012; The Public Health Act, Cap.242, 2022 | Yes | Food Act (No. 46 of 2017)  Food Quality Act (No. 402 of 1997)  Microbiology Regulation: Commission Regulation (EC) No 2073/2005 of 15 November 2005 on microbiological criteria for foodstuffs (; Food Regulation: Regulation (EC) No 178/2002 of the European Parliament and of the Council of 28 January 2002 laying down the general principles and requirements of food law, establishing the European Food Safety Authority and laying down procedures in matters of food safety ; Control Regulation: Regulation (EU) No 2017/625 of the European Parliament and of the Council of 15 March 2017 on official controls and other official activities performed to ensure the application of food and feed law, animal health and welfare rules, plant health and plant protection products; Commission Regulation (EC) No 2074/2005 of 5 December 2005 laying down detailed rules for the implementation of Regulation (EC) No 853/2004 of the European Parliament and of the Council as regards certain products and of official controls pursuant to Regulations (EC) No 854/2004 and (EC) No 882/2004 of the European Parliament and of the Council, derogating from Regulation (EC) No 852/2004 of the European Parliament and of the Council and amending Regulations (EC) No 853/2004 and (EC) No 854/2004 (latest version) |
|  |  |  |  |  |  |
| **P** | **Maximum residue limits (MRLs)** |  |  |  |  |
| **115** | Are maximum residue levels of pesticides and veterinary medicinal products in food under monitoring? | Yes | Standard Act, Cap.496, 2012 | Yes | Regulation (EC) No 470/2009 of the European Parliament and of the Council of 6 May 2009 laying down Community procedures for the establishment of residue limits of pharmacologically active substances in foodstuffs of animal origin, repealing Council Regulation (EEC) No 2377/90 and amending Directive 2001/82/EC of the European Parliament and of the Council and Regulation (EC) No 726/2004 of the European Parliament and of the Council.  Commission Regulation (EU) No 37/2010 of 22 December 2009 on pharmacologically active substances and their classification with regard to maximum residue limits in foodstuffs of animal origin.  Regulation 2018/470 laying down detailed rules on the maximum residue limit to be taken into account for the control of food of animal origin from animals treated in the EU in accordance with Article 11 of Directive 2001/82/EC. |
| **116** | Do all authorized antimicrobials have compulsory maximum residue limits? | Yes | Standard Act, Cap.496, 2012 | Yes | Regulation (EC) No 470/2009 of the European Parliament and of the Council of 6 May 2009 laying down Community procedures for the establishment of residue limits of pharmacologically active substances in foodstuffs of animal origin, repealing Council Regulation (EEC) No 2377/90 and amending Directive 2001/82/EC of the European Parliament and of the Council and Regulation (EC) No 726/2004 of the European Parliament and of the Council.  Commission Regulation (EU) No 37/2010 of 22 December 2009 on pharmacologically active substances and their classification with regard to maximum residue limits in foodstuffs of animal origin.  Regulation 2018/470 laying down detailed rules on the maximum residue limit to be taken into account for the control of food of animal origin from animals treated in the EU in accordance with Article 11 of Directive 2001/82/EC. |
| **117** | Are maximum residue limits in line with Codex standards? | Yes | Standard Act, Cap.496, 2012 | Yes | Regulation (EC) No 470/2009 of the European Parliament and of the Council of 6 May 2009 laying down Community procedures for the establishment of residue limits of pharmacologically active substances in foodstuffs of animal origin, repealing Council Regulation (EEC) No 2377/90 and amending Directive 2001/82/EC of the European Parliament and of the Council and Regulation (EC) No 726/2004 of the European Parliament and of the Council.  Commission Regulation (EU) No 37/2010 of 22 December 2009 on pharmacologically active substances and their classification with regard to maximum residue limits in foodstuffs of animal origin.  Regulation 2018/470 laying down detailed rules on the maximum residue limit to be taken into account for the control of food of animal origin from animals treated in the EU in accordance with Article 11 of Directive 2001/82/EC. |
|  |  |  |  |  |  |
| **Q** | **Environment, soil, and waste** |  |  |  |  |
| **118** | Is there a specific Act that regulates disposal of wastes (such as wastewater) from veterinary clinics and hospitals, or other agricultural enterprises that produce, use, or store antimicrobials? | Yes | Guidelines on veterinary medicinal products waste management | Yes | Act on environmental assessment of plans and programs (No. 4 of 2023)  Act on animal husbandry and use of manure and related matters (No. 338 of 2019) |
| **119** | Does the legislation provide on how to dispose of wastes from agricultural farms, abattoirs, quarantine areas, markets, and other premises where animals are kept or produced? | Except for agricultural farms | Veterinary Surgeons and Veterinary Paraprofessionals (Veterinary Medicines Directorate) Regulations,2015(L.N 174), 2015); meat act, public health act | Yes | Act on environmental assessment of plans and programs (No. 4 of 2023)  Act on animal husbandry and use of manure and related matters (No. 338 of 2019) |
| **120** | Are the relevant authorities empowered to authorize certain registration criteria or requirements for farms, or approve them to conduct other activities related to the environment? | Yes | National guidelines for safe management of healthcare waste, 2024 | Yes | Act on environmental assessment of plans and programs (No. 4 of 2023)  Act on animal husbandry and use of manure and related matters (No. 338 of 2019) |
| **121** | Before registration, are the farms required to present their environmental impact assessment? | Not required |  | Yes | Act on environmental assessment of plans and programs (No. 4 of 2023)  Act on animal husbandry and use of manure and related matters (No. 338 of 2019) |
| **122** | Are there national regulations that monitor the levels of antimicrobials in soil or prevent the pollution of water and soil including contaminations with antimicrobials? | No legislation available |  | Yes | Act on environmental assessment of plans and programs (No. 4 of 2023)  Act on animal husbandry and use of manure and related matters (No. 338 of 2019) |
| **123** | Are there provisions for the control of antimicrobial residues in wastes and effluents from mills, industries, farms, and other businesses requiring the gathering of animals? | No legislation available |  | Yes | Act on environmental assessment of plans and programs (No. 4 of 2023)  Act on animal husbandry and use of manure and related matters (No. 338 of 2019) |
| **R** | **Quality of water** |  |  |  |  |
| **124** | Is the quality of water used in aquatic and livestock farming regulated by the statute law? | Yes | Environmental Management and Co-Ordination Act, Cap.387, 2012 | Yes | Act on Water Resources Planning  Act on Watercourses (LBK no. 1217 of 2019)  Act on the use of fertilizers in agriculture and on nutrient-reducing measures (No. 338 of 2019).  Act amending the Environmental Protection Act,  Act on environmental permits of livestock, the Contaminated Soil |
| **125** | Does the law provide for the reuse of agricultural wastewater? | Yes | Environmental Management and Co-Ordination Act, Cap.387, 2012 | Yes | Act on the use of fertilizers in agriculture and on nutrient-reducing measures (No. 338 of 2019).  Act amending the Environmental Protection Act, Act on environmental permits of livestock, the Contaminated Soil  Act on animal husbandry and use of manure and related matters (No. 338 of 2019).  Order No. 1318 on commercial keeping of livestock, manure, silage.  Order No. 106 on Standards of Good Agricultural and Environmental Condition (GAEC) |
| **126** | Is antimicrobial use in aquatic farming controlled? | Yes | Guidelines for prudent use of antimicrobials in animals | Yes | Regulation on the use of vaccines and certain veterinary medicines for the prevention and control of certain listed animal diseases |
| **127** | Are the industrial and agricultural activities conducted in or around drinking water bodies regulated? | Yes | Environmental Management and Coordination Act, Cap.387, 2012 | Yes | Order No. 1318 on commercial keeping of livestock, manure, silage.  Order No. 106 on Standards of Good Agricultural and Environmental Condition (GAEC) |
| **128** | Are there provisions in the law to control the types of pollutants and discharges that are allowed to enter freshwater bodies? | Yes | Environmental Management and Coordination Act, Cap.387, 2012 | Yes | Order No. 1318 on commercial keeping of livestock, manure, silage.  Order No. 106 on Standards of Good Agricultural and Environmental Condition (GAEC)  Act on environmental permits of livestock, the Contaminated Soil |
|  |  |  |  |  |  |
| **S** | **Plant Health** |  |  |  |  |
| **129** | Is there a national body established for the prevention and control of AMR? | Yes | The Plant Protection Act, Cap.324, 2022 | Yes | Plants and Plant Health Act (No. 58 of 2020).  Plant Health Regulation (No. 1147 of 2022) |
| **130** | Are there any restrictions on supervision or oversight by an authorized person on the administration or use of AMs? | Yes | The Plant Protection Act, Cap.324, 2022 | Yes | Plants and Plant Health Act (No. 58 of 2020).  Plant Health Regulation (No. 1147 of 2022) |
|  |  |  |  |  |  |
| **T** | **Institutional Coordination** |  |  |  |  |
| **131** | Is there a national body established for prevention and control of AMR? | Yes | Kenya National Action Plan for the Prevention and Containment of Antimicrobial Resistance (2023-2027) | Yes | DANMAP (Danish Integrated Antimicrobial Resistance Monitoring and Research Program)  <https://www.danmap.org/> |
| **132** | Are the antimicrobial resistance coordination committee’s members, functions, and decision-making powers defined in the instrument? | Yes | Kenya National Action Plan for the Prevention and Containment of Antimicrobial Resistance (2023-2027) | Yes | DANMAP (Danish Integrated Antimicrobial Resistance Monitoring and Research Program)  <https://www.danmap.org/> |
| **133** | Is the information on funding NASIC provided? | No legislation available |  | Yes | DANMAP (Danish Integrated Antimicrobial Resistance Monitoring and Research Program)  <https://www.danmap.org/> |
| **134** | Are the private sector representatives included in NASIC? | Yes | Kenya National Action Plan for the Prevention and Containment of Antimicrobial Resistance (2023-2027) | Yes | DANMAP (Danish Integrated Antimicrobial Resistance Monitoring and Research Program)  <https://www.danmap.org/> |
| **135** | If the representatives of the private sector are included in the mechanism, are there ways to prevent possible conflicting interests? | Yes | Kenya National Action Plan for the Prevention and Containment of Antimicrobial Resistance (2023-2027) | Yes | DANMAP (Danish Integrated Antimicrobial Resistance Monitoring and Research Program)  <https://www.danmap.org/> |
| **136** | Is there a system that approves national legislation to be superior over the sub-national (decentralized) legislation about antimicrobials and antimicrobial resistance? | Yes | Kenya National Action Plan for the Prevention and Containment of Antimicrobial Resistance (2023-2027) | Yes | DANMAP (Danish Integrated Antimicrobial Resistance Monitoring and Research Program)  <https://www.danmap.org/> |
| **137** | Does the country have a prioritized research agenda within AMR? | No legislation available |  | Yes | DANMAP (Danish Integrated Antimicrobial Resistance Monitoring and Research Program)  <https://www.danmap.org/> |
| **138** | Is there a one health strategy against AMR from different relevant ministries or stakeholders? | Yes | Kenya National Action Plan for the Prevention and Containment of Antimicrobial Resistance (2023-2027)  Kenya Global Antimicrobial Resistance and Use Surveillance System (GLASS) Report 2022, 2022). | Yes | National One Health Strategy against Antibiotic Resistance |
| **U** | **The role of herd veterinarian** |  |  |  |  |
| **139** | Are veterinarians allowed to sell VMPs to make a profit? | No legislation available |  | No | Veterinarians Act (Veterinary Act) (LBK no. 64 of 19 January 2024)  Veterinary medicinal products and repeal of Directive 2001/82/EC (VMP) (EU Regulation 2019/6 of 11 December 2018) (consolidated version of 28 January 2022) |
| **140** | Are all types of farms obligated to have veterinary advisory service contracts between the veterinary practitioners and the farmers? | Yes | Guidelines for prudent use of antimicrobials in animals | Yes | Health advisory agreements for cattle herds (BEK 992 of May 25, 2021)  Guidance on health advisory agreements for cattle herds  Health advisory agreements for pig herds (BEK 991 of May 25, 2021)  Guidance on health advisory agreements for pig herds  Health advisory agreements for sheep and goat herds (BEK 993 of 25 May 2021)  Health advisory agreements for aquaculture companies (BEK 994 of 25 May 2021)  Mandatory health advice for mink herds (BEK 86 of 27 January 2023) |
| **141** | Are there treatment guidelines for specific hosts and/or diseases? | No legislation available |  | Yes | VetStat for veterinarians  <https://foedevarestyrelsen.dk/dyr/dyresundhed/laegemidler-til-dyr/vetstat/vetstat-for-dyrlaeger> |
| **142** | How often are the treatment guidelines revised? | No legislation available |  | Annually | Animal Health in Denmark report (2023) |
| **143** | Is AMR surveillance data taken into consideration when revising these guidelines? | No legislation available |  | Yes | Animal Health in Denmark report (2023) |
| **144** | Are there generally unannounced farm visits for inspection? | Yes | Guidelines for prudent use of antimicrobials in animals | Yes | Veterinarians Act (Veterinary Act) (LBK no. 64 of 19 January 2024) |
| **145** | Do veterinary practitioners revisit the livestock herds for complete registration when the gaps in gaps are noticed? | Yes | Guidelines for prudent use of antimicrobials in animals | Yes | Veterinarians Act (Veterinary Act) (LBK no. 64 of 19 January 2024) |
| **146** | Are there legal actions against the farmer such as issuance of fines in case he or she fails to comply with the rules within the stipulated time frame? | Yes | Animal Diseases act, 2006 | Yes | Veterinarians Act (Veterinary Act) (LBK no. 64 of 19 January 2024)  Animal owners' use of medicines for animals, etc. (Animal Owners' Executive Order) (BEK 645 of 31 May 2023)  Guidance on the Veterinary Medicinal Products Regulation and the Animal Owners Executive Order.pdf  ​ |
| **147** | Is there compulsory antimicrobial susceptibility testing before prescribing any AMs? | Yes | Guidelines for prudent use of antimicrobials in animals | Yes | Veterinarians Act (Veterinary Act) (LBK no. 64 of 19 January 2024)  Animal owners' use of medicines for animals, etc. (Animal Owners' Executive Order) (BEK 645 of 31 May 2023)  Guidance on the Veterinary Medicinal Products Regulation and the Animal Owners Executive Order.pdf |
| **148** | Is there mandatory showering, changing of clothes, handwashing, and disinfection for all persons involved in the chain of animal feed production, processing, and transportation to the end consumer? | Yes | Animal Diseases act, 2006 | Yes | Feed and feed companies (Feed Executive Order) (BEK 2227 of 30 November 2021); Regulation 2019/4 on the manufacture, placing on the market and use of medicated feed, amending Regulation (EC) No 183/2005 of the European Parliament and of the Council and repealing Council Directive 90/167/EEC; Feed and feed companies (Feed Guide) (VEJ 9006 of 10 January 2023) |
| **149** | Is it required that anyone who has interacted with pig herds must wash his or her hands and change clothing before leaving the herd? | Yes | Animal diseases act, 2006 (control of pig diseases) rules | Yes | Guidance on health advisory agreements for pig herds |
| **150** | Are persons working in the pig industry required to study a course in hygienic practices? | Yes | Guidelines for prudent use of antimicrobials in animals | Yes | Guidance on health advisory agreements for pig herds |
| **151** | Are the workers who deal with herds of pigs required to bathe at the end of the working day? Are individuals who often take short periods in pig stables required to put on disposable overalls? | Yes | Animal diseases act, 2006 (control of pig diseases) rules | Yes | Guidance on health advisory agreements for pig herds |
|  |  |  |  |  |  |
| **V** | **Monitoring program** |  |  |  |  |
| **152** | Is there an integrated antimicrobial use monitoring program? | No |  | Yes | DANMAP (Danish Integrated Antimicrobial Resistance Monitoring and Research Program)  <https://www.danmap.org/> |
| **153** | Is Antimicrobial use data centrally stored in one database that is accessible by all relevant institutions? | No |  | Yes | DANMAP (Danish Integrated Antimicrobial Resistance Monitoring and Research Program)  <https://www.danmap.org/> |
| **154** | Is there antimicrobial resistance data centrally stored in one database that is accessible by all relevant institutions? | Yes | Kenya National Action Plan for the Prevention and Containment of Antimicrobial Resistance (2023-2027)  Kenya Global Antimicrobial Resistance and Use Surveillance System (GLASS) Report 2022, 2022).  Veterinary Surgeons and Veterinary Paraprofessionals Act, 2015 (Veterinary Medicines Directorate Regulations) | Yes | DANMAP (Danish Integrated Antimicrobial Resistance Monitoring and Research Program)  <https://www.danmap.org/> |
| **155** | How is the monitoring program financed? | No legislation available |  | Yes | DANMAP (Danish Integrated Antimicrobial Resistance Monitoring and Research Program)  <https://www.danmap.org/> |
| **156** | Is there a national target for minimizing the use of antimicrobials in animals? | No legislation available |  | Yes | DANMAP (Danish Integrated Antimicrobial Resistance Monitoring and Research Program)  <https://www.danmap.org/> |
| **157** | How often is this target evaluated/revised? | No legislation available |  | Legislation available | DANMAP (Danish Integrated Antimicrobial Resistance Monitoring and Research Program)  <https://www.danmap.org/> |
| **158** | Is there benchmarking of the veterinarian’s prescription of antibiotics? | No legislation available |  | Yes | VetStat for veterinarians  <https://foedevarestyrelsen.dk/dyr/dyresundhed/laegemidler-til-dyr/vetstat/vetstat-for-dyrlaeger> |
| **159** | Are farmers required to attend courses on farming best practices, biosecurity, and prudent and reduced use of AMs including updates on changes to the legislation? | Yes | Guidelines for prudent use of antimicrobials in animals | Yes | VetStat for herd owners  <https://foedevarestyrelsen.dk/dyr/dyresundhed/laegemidler-til-dyr/vetstat/vetstat-for-besaetningsejere> |
